# Global, regional, and national burden of disease associated with low-fiber dietary patterns for colorectal cancer from 1990 to 2021: A systematic analysis for the global burden of disease 2021

**DOI:** 10.1097/MD.0000000000047982

**Published:** 2026-03-13

**Authors:** Ming Yin, Anmin Wang, Han Li, Chunyu Yang, Yuzhou Cai, Yujian Zeng

**Affiliations:** a Department of Intensive Care Medicine, The First Affiliated Hospital of Kunming Medical University, Kunming, Yunnan, China; b Department of General Surgery I, People’s Hospital of Guandu District, Kunming, China; c Department of Surgical Oncology, The First Affiliated Hospital of Kunming Medical University, Kunming, Yunnan, China; d Department of Gastroenterology, The First Affiliated Hospital of Kunming Medical University, Kunming, Yunnan, China; e Department of Gastrointestinal Surgery, The First Affiliated Hospital of Kunming Medical University, Kunming, Yunnan, China.

**Keywords:** colorectal cancer, DALY, dietary interventions, global burden, low-fiber diet

## Abstract

Low-fiber diets are a known risk factor for colorectal cancer (CRC). However, the burden of CRC associated with low-fiber intake across regions and age groups remains unclear. This study assesses the global, regional, and national burden of CRC due to low-fiber diets from 1990 to 2021. Using data from the Global Burden of Disease Study 2021, we applied comparative risk models to estimate CRC mortality and disability-adjusted life years associated with low-fiber diets across regions, age groups, and countries. The analysis showed significant disparities in CRC burden due to low-fiber diets. Low- and middle-income regions, particularly populations over 50 years old, bore the highest burden. The global burden has increased over time, particularly in regions undergoing dietary transitions. Targeted dietary interventions to increase fiber intake are essential to reduce the global CRC burden. Policymakers should focus on high-risk regions and populations to mitigate this preventable health issue.

## 1. Introduction

Colorectal cancer (CRC) remains one of the most prevalent and deadly cancers globally. Low dietary fiber intake is increasingly recognized as a key modifiable risk factor, highlighting the critical role of dietary habits in CRC development.^[1-3]^ Evidence from epidemiological and experimental studies suggests that a fiber-rich diet can help prevent CRC by improving intestinal health, enhancing bowel movements, and regulating gut microbiota.^[4-6]^ Despite this, dietary fiber intake remains insufficient in many regions, further exacerbating the CRC burden.^[7]^

The economic burden of CRC in 2021 was substantial, including high direct medical costs and indirect productivity losses.^[8-10]^ Diets low in fiber and high in meat consumption, commonly seen in Western regions, have been linked to higher CRC incidence. In contrast, traditionally high-fiber regions, such as parts of Asia and Africa, tend to have lower CRC rates.^[11-13]^ Socioeconomic status also plays a significant role, with lower-income individuals being more vulnerable to poor dietary habits, which worsens health disparities.^[14,15]^

Recent studies, such as those by Niu et al and Zhang et al,^[15,16]^ have examined the link between low-fiber diets and CRC in different populations. Niu et al explored the maternal and perinatal outcomes of CRC, further emphasizing the role of dietary factors in CRC outcomes. Similarly, Zhang et al discussed the pharmacological role of natural polyphenols in CRC, adding a layer of insight into dietary interventions for CRC prevention.

This study systematically quantifies the global burden of CRC associated with low-fiber diets from 1990 to 2021. The findings provide valuable insights for public health strategies, including promoting fiber-rich diets, implementing healthy eating policies, and supporting community-based dietary interventions.^[17,18]^

## 2. Materials and methods

### 2.1. Overview

We utilized the Global Burden of Disease (GBD) 2021 database to estimate the number of deaths and disability-adjusted life years (DALYs) associated with low-fiber diet patterns. Low-fiber diet exposure was defined as an average daily fiber intake of <25 g, in line with the GBD model’s criteria. This threshold was selected based on international dietary guidelines and reflects dietary patterns observed in various global regions.

To estimate the burden of CRC associated with low-fiber diets, the study used the comparative risk modeling framework employed by the GBD 2021 study. This framework integrates a variety of risk factors, including dietary intake levels, socioeconomic factors, and the demographic characteristics of each region. The GBD model assumes that populations with lower fiber intake levels are at a higher risk for developing CRC, and thus the burden of CRC associated with low-fiber diets is modeled based on these assumptions.

We employed linear regression models to estimate the annual percentage change (estimated annual percentage change [EAPC]) in CRC mortality and DALYs due to low-fiber diets from 1990 to 2021. The model’s estimates were cross-tabulated with data from various regions and sociodemographic index (SDI) to identify regional trends and disparities. Additionally, Spearman’s correlation analysis was used to assess the relationship between low-fiber diet exposure, socioeconomic development, and CRC outcomes.

The disease modeling – meta-regression framework, used in the GBD model, helps to estimate mortality rates and remission rates for diseases. In this study, additional adjustments were made using the meta-regression-Bayesian, regularized, trimmed tool to correct for any potential systematic biases, ensuring the accuracy of our estimates. It is important to note that GBD modeling typically assigns a value of zero for non-existent mortality or remission rates, following standard practice.

### 2.2. Study data

This study utilizes data from the GBD 2021 study, which comprehensively assessed health losses attributable to diseases, injuries, and risk factors across 204 countries and regions from 1990 to 2021. The GBD study employs consistent and transparent methodologies, integrating vital registration systems, disease registry systems, survey data, and census data to estimate health outcomes and risk exposures.

Following the GBD comparative risk assessment framework, dietary fiber intake was modeled as continuous exposure (g/d) using a residual method calibrated to a 2000 kcal/d diet. The theoretical minimum risk exposure level (TMREL) for fiber was approximately 23.5 g/d; exposure below TMREL was associated with population-level risk. Dose-response functions were derived via meta-regression-Bayesian, regularized, trimmed methods, allowing nonlinear curves with spline constraints and inter-study heterogeneity.

Practical note for readers: We report results using the GBD standard CRC cause-specific × low-fiber diet interaction, where “attributable” burden strictly denotes the population-attributable fraction calculated based on TMREL, not inferences of causality from this study design.

Due to data availability, GBD only includes individuals aged 25 years and older in this category; thus, the study focuses on this age group. Subjects were stratified into 15 age cohorts (5-year intervals) to examine specific trends in age-specific CRC burden attributable to low-fiber diets.

### 2.3. Statistical analysis

#### 2.3.1. Estimation of disease burden

We used data from the GBD 2021 study to quantify the burden of CRC attributable to low-fiber dietary patterns. The burden was assessed in terms of deaths and DALYs in 2021, stratified by several factors, including age, sex, SDI, and GBD regions and countries.

Age-standardized rates (ASRs; age-standardized mortality rates [ASMR] and age-standardized death rates [ASDR]) were calculated to account for differences in the age distribution across populations. Age-standardization allows for comparisons across regions with different demographic structures, ensuring that differences in CRC burden are not merely a result of variations in population age distributions.

The SDI, which integrates factors like income, education, and fertility rates, is used to categorize countries. We used SDI as a proxy for socioeconomic development, and stratifying the data by SDI allows us to examine how socioeconomic factors might influence the burden of CRC linked to low-fiber diets.

#### 2.3.2. Temporal trend analysis and EAPC calculation

To explore how the burden of CRC attributable to low-fiber diets has changed over time, we conducted a temporal trend analysis. This was done by calculating the EAPC for ASMR and ASDR from 1990 to 2021.

The EAPC is calculated using a linear regression model, which fits a straight line to the time series data. This allows us to quantify the rate of change in CRC burden over time. Specifically, the regression model estimates the slope of the line, which represents the annual percentage change in the outcome variable (e.g., CRC mortality or DALYs).

This calculation gives us a measure of the trend’s direction (whether it’s increasing or decreasing) and magnitude. A negative EAPC indicates a decline in CRC burden, while a positive EAPC indicates an increase.

#### 2.3.3. Hierarchical cluster analysis

To detect patterns of change in CRC burden across different regions, we performed hierarchical cluster analysis on the EAPC values calculated earlier. Hierarchical clustering is an unsupervised machine learning technique that groups regions with similar trends.

The goal of this analysis is to identify regions that have experienced similar trends in CRC burden over the study period (1990–2021). By clustering regions with similar temporal changes, we can identify global patterns in CRC burden related to low-fiber diets.

Agglomerative clustering was used, where each region starts as its own cluster, and pairs of clusters are merged based on similarity until all regions are grouped into a hierarchy. The similarity between clusters is calculated using a distance metric (usually Euclidean distance), which quantifies the difference in EAPC values between regions.

Dendrograms are generated as a result of this analysis, visually representing the hierarchical structure of clusters. This allows us to easily identify clusters of regions with similar trends, which can be further analyzed for insights into global patterns in CRC burden.

#### 2.3.4. Frontier analysis

To benchmark the performance of each region in reducing the burden of CRC attributable to low-fiber diets, we used frontier analysis. This technique compares the performance of each region with that of the “most efficient” region = the one that has minimized the burden of CRC to the greatest extent.

Frontier analysis uses a non-parametric approach, often implemented through Data Envelopment Analysis) or Stochastic Frontier Analysis, to assess the relative efficiency of regions in terms of CRC burden reduction. The “frontier” is the boundary that represents the best possible performance, and the closer a region’s performance is to this frontier, the more efficient it is considered in reducing CRC burden.

By identifying the regions that are closest to the frontier, we can provide examples of best practices and identify regions that could potentially improve by emulating these top-performing regions.

#### 2.3.5. Correlation with sociodemographic index (SDI) and HDI

To explore the relationship between the burden of CRC due to low-fiber diets and socioeconomic development, we calculated the Spearman rank correlation between EAPC values, ASRs of CRC-related deaths (ASMR), and the human development index (HDI) in 2021.

The Spearman correlation was used instead of Pearson’s correlation because the data did not follow a normal distribution. Spearman’s correlation assesses the strength and direction of the monotonic relationship between 2 variables = in this case, the temporal trends in CRC burden (EAPC) and HDI components (life expectancy, income, and education).

The correlation coefficient (ρ\rhoρ) ranges from −1 to 1, where values closer to 1 indicate a strong positive relationship, values closer to −1 indicate a strong negative relationship, and values near 0 suggest no correlation.

The HDI was chosen because it provides a comprehensive measure of socioeconomic development and reflects the broader social determinants of health that could influence the burden of CRC related to low-fiber diets.

#### 2.3.6. Statistical software

All statistical analyses were performed using R software (version 4.3.2), a widely used and powerful environment for statistical computing and data visualization. The analyses were conducted using the following R packages: “dplyr” for data manipulation and preparation.”ggplot2” for data visualization”cluster” for hierarchical clustering”corrr” for Spearman’s correlation analysis”lm” and “stats” for linear regression and EAPC calculation. The reproducibility of the analyses was ensured by using standardized data processing pipelines, making the results easily replicable for future studies.

## 3. Results

### 3.1. Disease burden due to colorectal cancer associated with a low-fiber diet pattern in 2021

In 2021, an estimated 9689 deaths globally were associated with a low-fiber diet contributing to CRC, accounting for 0.02% of total global deaths. The death estimates ranged from 4410 to 14,808. The age-standardized mortality rate was 0.27/1,00,000 (95% uncertainty interval [UI]: 0.07–0.24). Additionally, the DALYs lost due to low-fiber diets were estimated at 3,05,676 years (95% UI: 1,35,089–4,69,863), representing 0.01% of total global DALYs. The corresponding age-standardized DALY rate was 3.58/1,00,000 people (95% UI: 1.58–5.5). For further details, refer to Supplementary Table S2, Supplemental Digital Content, https://links.lww.com/MD/R530.

**Figure 1.**
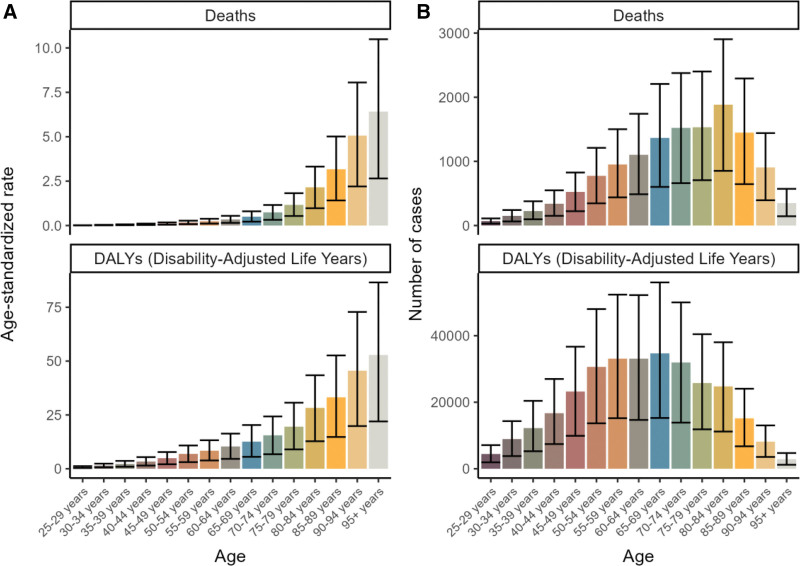
Burden of colorectal cacer low-fiber diet across age groups in 2021. (A) Age-standardized death rate and age-standardized DALY rate by age group. (B) Number of deaths and number of DALYs by age group. DALY = disability-adjusted life year.

Figure 1A shows the age-standardized trends in mortality and DALYs for various age groups in 2021, with both rates increasing with age. The highest number of deaths was observed in the 80 to 84 age group, while DALYs peaked in the 65 to 69 age group before showing a decline (Fig. 1A, B, Tables S1–S2, Supplemental Digital Content, https://links.lww.com/MD/R530).

**Figure 2.**
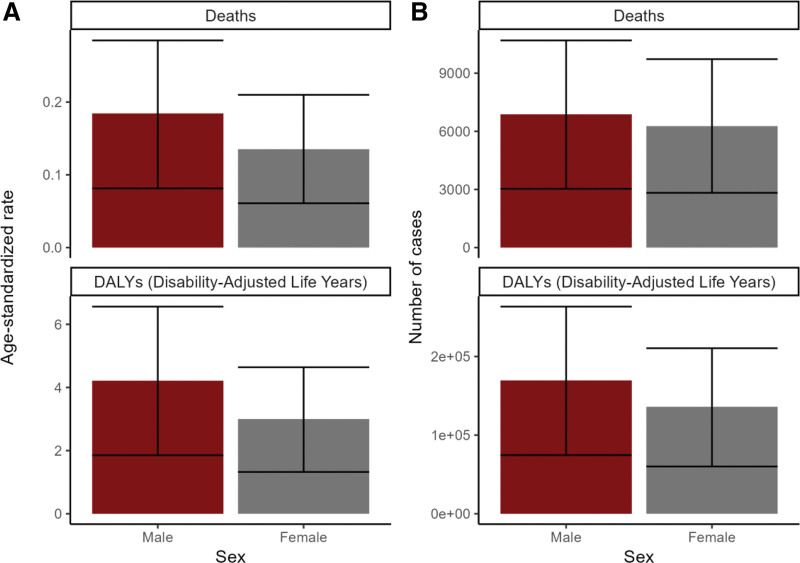
Burden of colorectal cancer attributable to a low-fiber diet by sex in 2021. (A) Age-standardized death rate and age-standardized DALY rate by sex. (B) Number of deaths and number of DALYs by sex. DALY = disability-adjusted life year.

The data also revealed that, compared to women, men had 10% higher mortality and 25% more DALYs due to low-fiber diets in 2021. Specifically, the age-standardized mortality and DALY rates for men were 29% and 40% higher than for women, respectively (Fig. 2A, B, Tables S1–S2, Supplemental Digital Content, https://links.lww.com/MD/R530).

**Figure 3.**
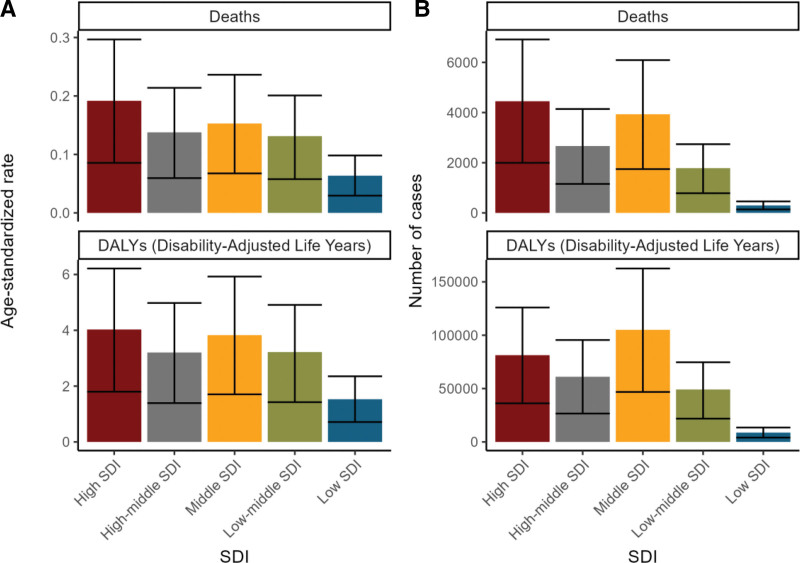
Burden of colorectal cancer attributable to a low-fiber diet across SDI regions in 2021. (A) Age-standardized death rate and age-standardized DALY rate by SDI region. (B) Number of deaths and number of DALYs by SDI region. DALY = disability-adjusted life year, SDI = sociodemographic index.

**Figure 4.**
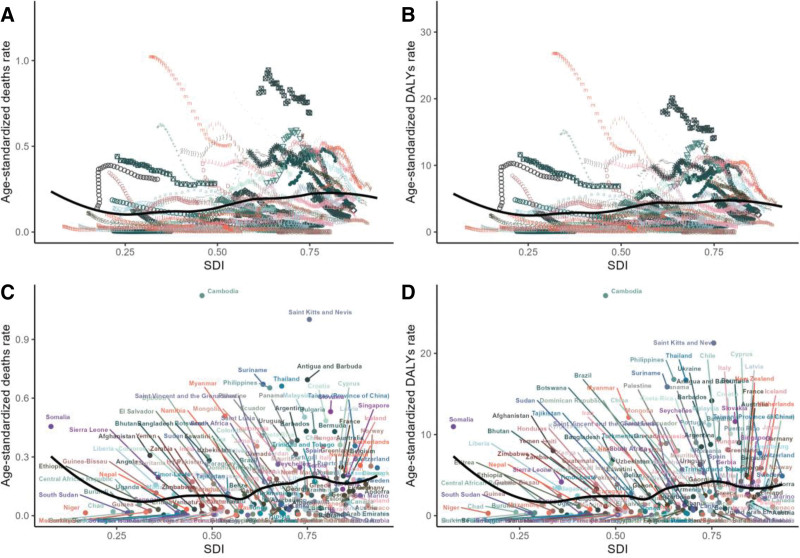
Relationship between the burden of colorectal cancer attributable to a low-fiber diet and SDI across countries and regions, 1990-2020. (A) Regional trajectories of age-standardized death rates by SDI. (B) Regional trajectories of age-standardized DALY rates by SDI. (C) National distribution of age-standardized death rates by SDI. (D) National distribution of age-standardized DALY rates by SDI. DALY = disability-adjusted life year, SDI = sociodemographic index.

The analysis by SDI area revealed that the high SDI area had the highest number of deaths, amounting to 4450, and the highest age-standardized mortality rate. In contrast, the DALYs were highest in the medium SDI areas, reaching 1,05,062 (see Fig. 3A, B and Supplementary Tables S1 and S2, Supplemental Digital Content, https://links.lww.com/MD/R530). It is notable that there is considerable heterogeneity in the distribution of disease burden across countries and regions with disparate SDI levels. Figure 4 illustrates an intriguing phenomenon: a U-shaped relationship exists between the age-standardized ratio of deaths and DALYs and SDI. Specifically, when the SDI is below 0.50, the age-standardized mortality rate increases with increasing SDI. However, when the SDI exceeds 0.50, the age-standardized mortality rate decreases with increasing SDI. This trend reflects the intricate, nonlinear relationship between SDI and health outcomes.

**Figure 5.**
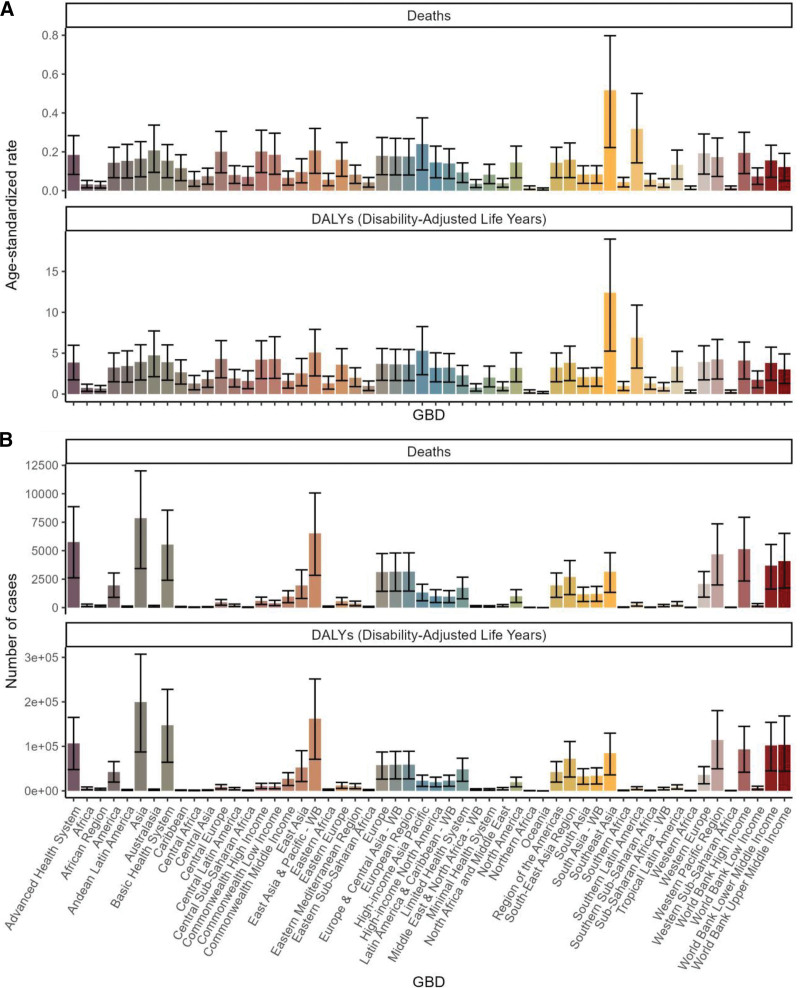
Burden of colorectal cancer attributable to a low-fiber diet across GBD regions in 2021. (A) Age-standardized death rate and age-standardized DALY rate by region. (B) Number of deaths and number of DALYs by region. DALY = disability-adjusted life year, GBD = Global Burden of Disease.

A regional analysis based on the GBD framework revealed that Asia had the highest number of deaths due to low-fiber diets and CRC, with a total of 7856 cases [95% UI: 3432–12,008]. Furthermore, the region exhibited the highest number of DALYs, at 1,99,582 [95% UI: 87,235–3,07,130]. In contrast, Oceania exhibited the lowest incidence of mortality from this health problem, with almost zero deaths. It is noteworthy that Southeast Asia exhibited the highest age-standardized mortality rate (0.16, 95% UI: 0.07 to 0.25) and a significantly increasing trend in age-standardized DALYs (12.4, 95% UI: 5.25 to 18.96; Fig. 5A, B, Tables 1 and S1–S2, Supplemental Digital Content, https://links.lww.com/MD/R530).

**Table 1:** Global number of colorectal cancer cases and age-standardized death rates due to a low-fiber diet in 1990 and 2021, aggregated by SDI region, GBD region, and trends from 1990 to 2021.

Characteristic	1990		2021		1990–2021
	Number of deaths cases (95% UI)	The age-standardized deaths rate/1,00,000 (95% UI)	Number of deaths cases (95% UI)	The age-standardized deaths rate/1,00,000 (95% UI)	EAPC (95% CI)
Global	9689 (4410–14,808)	0.27 (0.12–0.42)	13,145 (5762–20,265)	0.16 (0.07–0.24)	−1.86 (−1.9 to −1.82)
SDI region					
High-middle SDI	2002 (917–3117)	0.22 (0.1–0.34)	2666 (1154–4141)	0.14 (0.06–0.21)	−1.63 (−1.82 to −1-1.45)
High SDI	3927 (1747–5986)	0.36 (0.16–0.54)	4450 (1995–6913)	0.19 (0.09–0.3)	−1.97 (−2.05 to −1.89)
Low-middle SDI	1043 (467–1622)	0.18 (0.08–0.28)	1784 (784–2738)	0.13 (0.06–0.2)	−1.25 (−1.38 to −1.11)
Low SDI	153 (68–236)	0.07 (0.03–0.12)	298 (138–458)	0.06 (0.03–0.1)	−0.74 (−0.96 to −0.53)
Middle SDI	2554 (1182–3972)	0.26 (0.12–0.41)	3931 (1746–6090)	0.15 (0.07–0.24)	−1.9 (−1.97 to −1.84)
GBD region					
Advanced health system	4695 (2078–7184)	0.29 (0.13–0.45)	5759 (2616–8867)	0.18 (0.08–0.28)	−1.58 (−1.62 to −1.53)
Africa	107 (49–169)	0.04 (0.02–0.07)	190 (85–308)	0.03 (0.01–0.05)	−1 (−1.04 to −0.95)
African Region	83 (38–130)	0.04 (0.02–0.07)	134 (59–211)	0.03 (0.01–0.05)	−1.25 (−1.31 to −1.18)
America	2029 (921–3101)	0.35 (0.16–0.53)	1948 (903–3040)	0.14 (0.07–0.22)	−2.76 (−2.95 to −2.57)
Andean Latin America	36 (15–55)	0.19 (0.08–0.29)	88 (38–137)	0.15 (0.07–0.24)	−0.47 (−0.64 to −0.29)
Asia	4946 (2325–7765)	0.26 (0.12–0.41)	7856 (3432–12,008)	0.16 (0.07–0.25)	−1.59 (−1.67 to −1.51)
Australasia	102 (46–162)	0.44 (0.2–0.7)	118 (53–195)	0.21 (0.09–0.34)	−2.9 (−3.15 to −2.65)
Basic health system	3883 (1827–6143)	0.28 (0.13–0.44)	5534 (2403–8562)	0.15 (0.07–0.24)	−2.02 (−2.09 to −1.94)
Caribbean	60 (27–94)	0.24 (0.11–0.38)	62 (28–99)	0.12 (0.05–0.18)	−2.51 (−2.77 to −2.25)
Central Africa	7 (3–11)	0.03 (0.01–0.05)	32 (13–57)	0.06 (0.02–0.1)	2.3 (1.82–2.79)
Central Asia	71 (30–107)	0.15 (0.07–0.23)	57 (25–88)	0.07 (0.03–0.12)	−3.19 (−3.65 to −2.73)
Central Europe	302 (136–466)	0.21 (0.1–0.33)	464 (212–711)	0.2 (0.09–0.31)	−0.54 (−0.91 to −0.17)
Central Latin America	61 (27–92)	0.08 (0.03–0.12)	196 (91–310)	0.08 (0.04–0.13)	0.22 (0.08–0.37)
Central Sub-Saharan Africa	10 (4–16)	0.05 (0.02–0.08)	33 (14–59)	0.07 (0.03–0.12)	1.19 (0.88–1.5)
Commonwealth high income	679 (299–1068)	0.44 (0.2–0.69)	590 (274–920)	0.2 (0.09–0.31)	−2.5 (−2.69 to −2.3)
Commonwealth low income	217 (100–345)	0.26 (0.12–0.41)	391 (176–641)	0.18 (0.08–0.3)	−1.23 (−1.37 to −1.09)
Commonwealth middle income	551 (248–863)	0.09 (0.04–0.15)	969 (430–1481)	0.07 (0.03–0.1)	−1.37 (−1.55 to −1.19)
East Asia	2180 (961–3566)	0.27 (0.12–0.44)	1942 (799–3318)	0.1 (0.04–0.16)	−3.43 (−3.53 to −3.32)
East Asia and Pacific-WB	4185 (1958–6580)	0.34 (0.16–0.53)	6528 (2833–10,071)	0.21 (0.09–0.32)	−1.63 (−1.69 to −1.56)
Eastern Africa	50 (22–80)	0.08 (0.04–0.13)	82 (37–135)	0.06 (0.03–0.09)	−1.45 (−1.57 to −1.34)
Eastern Europe	375 (173–584)	0.14 (0.06–0.22)	567 (261–889)	0.16 (0.07–0.25)	−0.7 (−1.3 to −0.09)
Eastern Mediterranean Region	144 (64–223)	0.09 (0.04–0.14)	350 (160–565)	0.08 (0.04–0.13)	−0.31 (−0.52 to −0.1)
Eastern Sub-Saharan Africa	38 (17–61)	0.06 (0.03–0.09)	65 (28–105)	0.04 (0.02–0.07)	−1.32 (−1.45 to −1.19)
Europe	2589 (1149–3943)	0.25 (0.11–0.39)	3125 (1429–4748)	0.18 (0.08–0.27)	−1.35 (−1.5 to −1.2)
Europe and Central Asia-WB	2637 (1169–4014)	0.25 (0.11–0.38)	3155 (1444–4795)	0.18 (0.08–0.27)	−1.39 (−1.54 to −1.23)
European Region	2643 (1171–4023)	0.25 (0.11–0.38)	3165 (1449–4810)	0.18 (0.08–0.27)	−1.39 (−1.55 to −1.23)
High-income Asia Pacific	383 (161–599)	0.21 (0.09–0.33)	1324 (601–2055)	0.24 (0.11–0.37)	0.78 (0.6–0.96)
High-income North America	1534 (704–2359)	0.43 (0.2–0.65)	992 (448–1577)	0.15 (0.07–0.23)	−3.47 (−3.66 to −3.28)
Latin America and Caribbean-WB	502 (223–758)	0.21 (0.09–0.32)	967 (432–1490)	0.14 (0.06–0.22)	−1.15 (−1.25 to −1.05)
Limited health system	1067 (483–1668)	0.14 (0.07–0.22)	1740 (777–2674)	0.09 (0.04–0.14)	−1.63 (−1.78 to −1.48)
Middle East and North Africa-WB	45 (20–71)	0.04 (0.02–0.07)	108 (49–173)	0.04 (0.02–0.06)	−0.59 (−0.69 to −0.48)
Minimal health system	34 (15–55)	0.07 (0.03–0.1)	97 (42–163)	0.08 (0.04–0.14)	0.63 (0.24–1.01)
North Africa and Middle East	66 (29–106)	0.04 (0.02–0.07)	162 (74–267)	0.04 (0.02–0.06)	−0.51 (−0.61 to −0.42)
North America	1534 (704–2359)	0.43 (0.2–0.65)	992 (449–1578)	0.15 (0.07–0.23)	−3.47 (−3.66 to −3.28)
Northern Africa	11 (5–18)	0.02 (0.01–0.04)	18 (7–31)	0.01 (0.01–0.02)	−1.61 (−1.94 to −1.28)
Oceania	0 (0–1)	0.02 (0.01–0.03)	0 (0–1)	0.01 (0–0.01)	−2.4 (−2.75 to −2.05)
Region of the Americas	2029 (921–3101)	0.35 (0.16–0.53)	1948 (903–3040)	0.14 (0.07–0.22)	−2.76 (−2.95 to −2.57)
South-East Asia Region	1525 (698–2383)	0.23 (0.11–0.36)	2691 (1141–4135)	0.16 (0.07–0.25)	−1.51 (−1.63 to −1.39)
South Asia	664 (307–1067)	0.12 (0.06–0.2)	1166 (533–1795)	0.08 (0.04–0.13)	−1.44 (−1.6 to −1.28)
South Asia-WB	678 (313–1087)	0.12 (0.06–0.19)	1205 (548–1860)	0.08 (0.04–0.13)	−1.4 (−1.57 to −1.23)
Southeast Asia	1524 (691–2296)	0.63 (0.29–0.95)	3152 (1338–4822)	0.52 (0.22–0.8)	−0.8 (−0.86 to −0.73)
Southern Africa	16 (7–25)	0.04 (0.02–0.07)	36 (16–57)	0.04 (0.02–0.07)	0.05 (−0.06 to 0.17)
Southern Latin America	198 (89–299)	0.46 (0.21–0.69)	284 (127–446)	0.32 (0.14–0.5)	−0.48 (−0.71 to −0.25)
Southern Sub-Saharan Africa	10 (4–15)	0.04 (0.02–0.06)	30 (13–46)	0.06 (0.02–0.09)	1.23 (0.97–1.49)
Sub-Saharan Africa - WB	95 (43–151)	0.05 (0.02–0.08)	171 (76–276)	0.04 (0.02–0.06)	−0.87 (−0.91 to −0.83)
Tropical Latin America	148 (66–225)	0.18 (0.08–0.27)	338 (153–529)	0.13 (0.06–0.21)	−1.32 (−1.55 to −1.1)
Western Africa	23 (10–35)	0.03 (0.01–0.05)	21 (9–34)	0.01 (0.01–0.02)	−2.84 (−3.14 to −2.53)
Western Europe	1900 (838–2902)	0.32 (0.14–0.48)	2079 (919–3168)	0.19 (0.09–0.29)	−1.58 (−1.68 to −1.49)
Western Pacific Region	3183 (1464–5030)	0.3 (0.14–0.48)	4678 (1988–7360)	0.17 (0.07–0.27)	−1.78 (−1.85 to −1.71)
Western Sub-Saharan Africa	25 (11–38)	0.03 (0.01–0.05)	23 (10–39)	0.01 (0.01–0.02)	−2.8 (−3.1 to −2.51)
World Bank high income	4306 (1901–6594)	0.33 (0.15–0.51)	5139 (2336–7936)	0.19 (0.09–0.3)	−1.67 (−1.75 to −1.59)
World Bank low income	93 (40–147)	0.07 (0.03–0.11)	219 (97–355)	0.07 (0.03–0.12)	−0.16 (−0.34 to 0.03)
World Bank lower middle income	1967 (895–3009)	0.2 (0.09–0.3)	3690 (1629–5545)	0.16 (0.07–0.23)	−1.03 (−1.19 to −0.88)
World Bank upper middle income	3313 (1551–5292)	0.24 (0.11–0.38)	4081 (1717–6521)	0.12 (0.05–0.19)	−2.37 (−2.5 to −2.25)

CI = confidence interval, EAPC = estimated annual percentage change, GBD = Global Burden of Disease, SDI = sociodemographic Index, UI = uncertainty interval.

In 2021, significant geographical discrepancies were observed in the burden of CRC associated with low-fiber diets across countries. Cambodia had the highest age-standardized mortality rate at 1.12/1,00,000 (95% UI: 0.52–1.83), followed by Saint Kitts and Nevis (1.00, 95% UI: 0.45–1.59) and Vietnam (0.72, 95% UI: 0.32–1.17). Similarly, Cambodia led in age-standardized DALYs, with 27.12 years per 1,00,000 (95% UI: 12.59–44.31), followed by Saint Kitts (21.27, 95% UI: 9.27–34.29) and Vietnam (17.41, 95% UI: 7.87–28.31).

**Figure 6.**
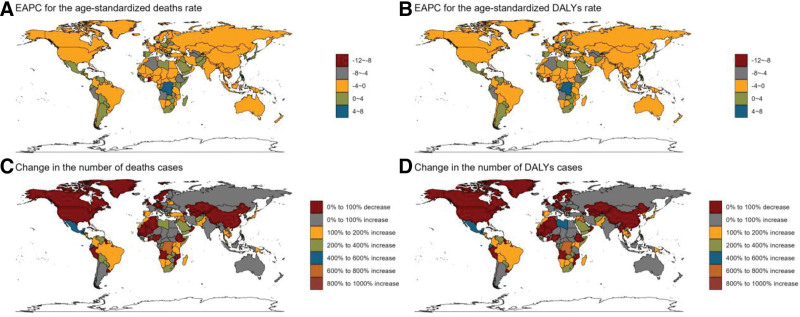
Number of deaths and disability-adjusted life years associated with low-fiber diet-attributable colorectal cancer and age-standardized rate by country and region, 2021. (A) EAPC for the age-standardized deaths rate. (B) EAPC for the age-standardized DALYs rate. (C) Change in the number of deaths cases. (D) Change in the number of DALYs cases. DALY = disability-adjusted life year, EAPC = estimated annual percentage change.

China had the highest absolute number of deaths, with 1739 (95% UI: 693–3024), surpassing the United States (914, 95% UI: 407–1443) and Japan (825, 95% UI: 360–1337). China also accounted for the highest burden of DALYs at 481,000 years (95% UI: 1,88,670–8,47,610), followed by Indonesia (2,00,000 years, 95% UI: 86,090–332,140) and the United States (18,261 years, 95% UI: 8373–28,368). These findings highlight the relationship between population size and disease burden, emphasizing the need for region-specific prevention strategies (Fig. 6A–D, Tables 1 and S1–S2, Supplemental Digital Content, https://links.lww.com/MD/R530).

### 3.2. Time trends in the burden of disease associated with colorectal cancer associated with a low-fiber diet from 1990 to 2021

**Figure 7.**
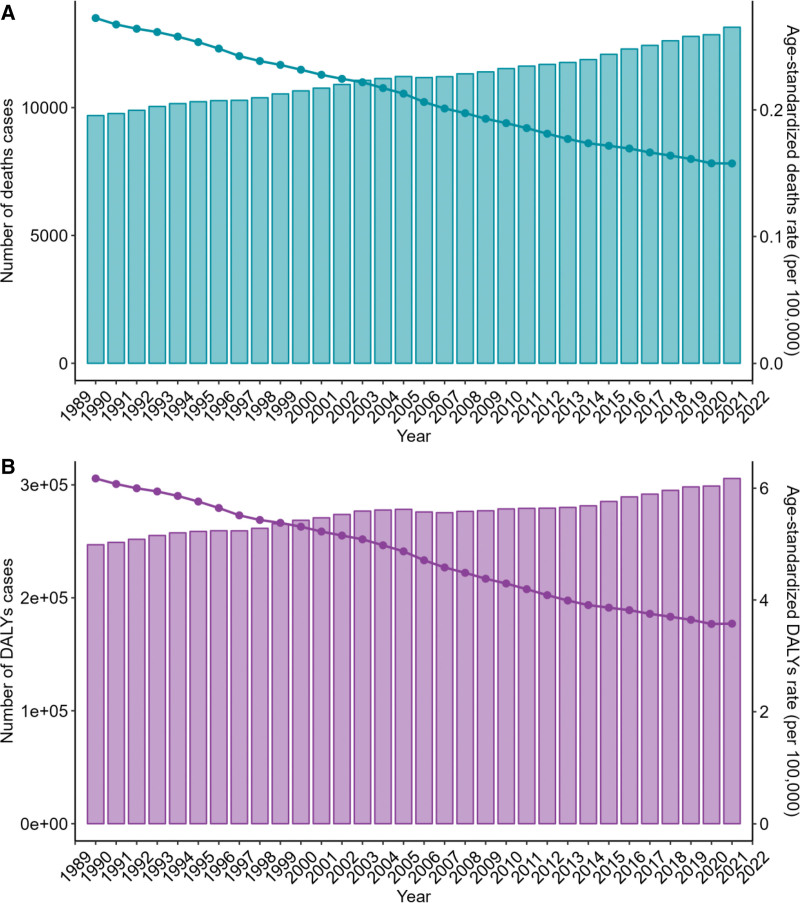
Global trends in the burden of colorectal cancer attributable to a low-fiber diet, 1990-2021. (A) Trends in the number of deaths and the age-standardized death rate. (B) Trends in the number of DALYs and the age-standardized DALY rate. DALY = disability-adjusted life year.

**Figure 8.**
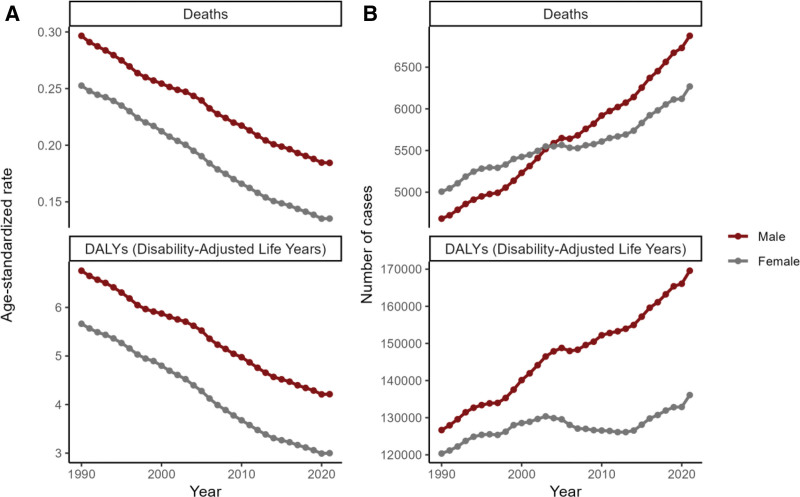
Sex-specific global trends in the burden of colorectal cancer attributable to a low-fiber diet, 1990-2021. (A) Trends in age-standardized death rates and age-standardized DALY rates by sex. (B) Trends in the number of deaths and number of DALYs by sex. DALY = disability-adjusted life year.

From 1990 to 2021, the global number of deaths from CRC associated with a low-fiber diet increased by 35.67%, from 9689 to 13,145. Similarly, DALYs rose by 23.75%. However, age-standardized mortality and DALYs showed a downward trend, with mortality decreasing by 42.46% and DALYs by 43.4% (Figs. 7 and 8, Tables 1 and S1–S2, Supplemental Digital Content, https://links.lww.com/MD/R530).

**Figure 9.**
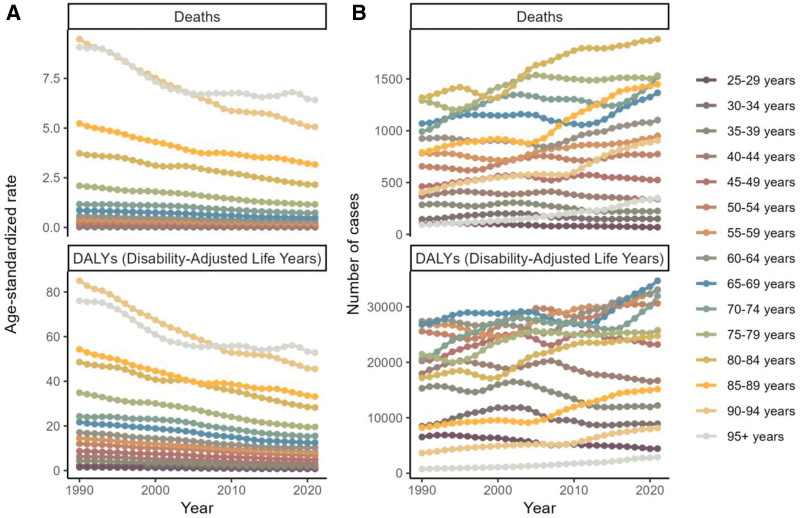
Age-specific global trends in the burden of colorectal cancer attributable to a low-fiber diet, 1990-2021. (A) Trends in age-standardized death rates and age-standardized DALY rates by age group. (B) Trends in the number of deaths and number of DALYs by age group. DALY = disability-adjusted life year.

The trends for men and women were consistent with the overall population, as shown in Figure 8 and Tables S1–S2, Supplemental Digital Content, https://links.lww.com/MD/R530. An upward trend in deaths and DALYs was observed across all age groups, except for the under-30 and 35 to 44 age groups (Fig. 9, Tables S1–S2, Supplemental Digital Content, https://links.lww.com/MD/R530).

**Figure 10.**
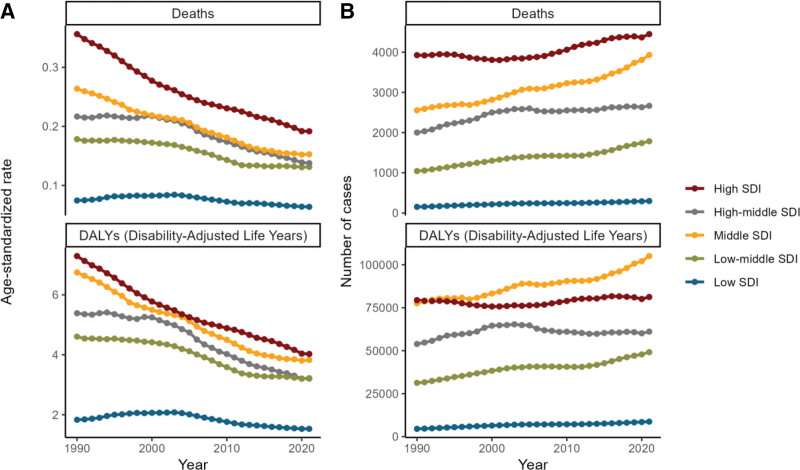
Global trends in the burden of colorectal cancer attributable to a low-fiber diet by SDI region, 1990-2021. (A) Trends in age-standardized death rates and age-standardized DALY rates by SDI region. (B) Trends in the number of deaths and number of DALYs by SDI region. DALY = disability-adjusted life year, SDI = sociodemographic index.

Regional analysis based on the SDI revealed that all SDI regions experienced a decline in age-standardized mortality and DALY rates, with the sharpest decline in the medium SDI region. The low and lower-middle SDI regions showed stable mortality trends, while the upper-middle SDI region saw an initial increase followed by a decline. DALY trends mirrored these patterns, indicating disparities in disease burden across SDI regions (Fig. 10, Tables 1 and S1–S2, Supplemental Digital Content, https://links.lww.com/MD/R530).

**Figure 11.**
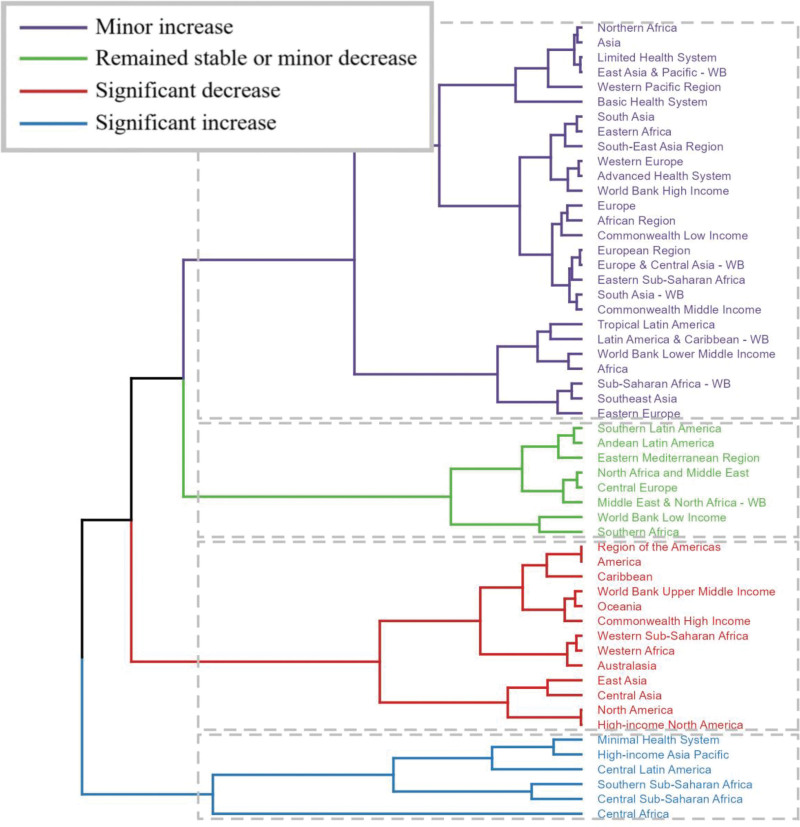
Results of the cluster analysis based on EAPC values for age-standardized mortality and DALYs rates associated with colorectal cancer associated with a low-fiber diet from 1990 to 2021. EAPC = estimated annual percentage change, DALY = disability-adjusted life year, SDI = sociodemographic index.

Significant regional disparities were also noted in the GBD regions. Hierarchical cluster analysis identified regions with similar trends in disease burden. Notable increases in age-standardized mortality and DALYs were observed in regions with weaker health systems, such as Central America, Central Africa, and parts of Sub-Saharan Africa. Conversely, these indicators declined in upper-middle-income regions, high-income Commonwealth countries, and parts of North America and Australasia (Fig. 11, Tables 1 and S2, Supplemental Digital Content, https://links.lww.com/MD/R530).

From 1990 to 2021, several countries and regions saw a significant increase in CRC deaths due to a low-fiber diet. Iraq, the Democratic Republic of the Congo, and the Republic of Korea had the largest increases, with mortality rates rising by 1200%, 700%, and 486.59%, respectively. In contrast, Cuba, Azerbaijan, and Kyrgyzstan experienced the most significant decreases in deaths, with reductions of 77.42%, 50%, and 50%, respectively.

Similarly, Burundi, Iraq, and the Democratic Republic of the Congo showed the largest increases in DALYs, with rises of 850%, 795.12%, and 748.78%, respectively. Countries with the greatest reductions in DALYs were Ghana, Cuba, and Senegal, with decreases of 90.91%, 82.81%, and 69.84%, respectively.

ASMR also showed substantial regional variation. Burundi, the Democratic Republic of the Congo, and Iraq saw notable increases, with EAPC of 4.42 (95% confidence interval [CI]: 3.73–5.12), 4.16 (95% CI: 3.3–5.03), and 3.09 (95% CI: 2.33–3.85), respectively. On the other hand, Ghana, Cuba, and Equatorial Guinea demonstrated significant declines in mortality rates, with EAPC values of −10.48 (95% CI: −11.66 to −9.29), −8.08 (95% CI: −9.28 to −6.87), and −7.06 (95% CI: −8.17 to −5.94), respectively.

**Figure 12.**
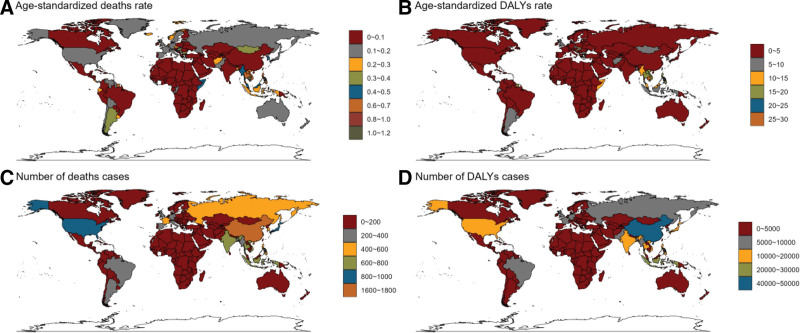
Trends in the number of deaths, disability-adjusted life years and age-standardized rates associated with colorectal cancer due to a low-fiber diet worldwide, grouped by country region. (A) Age-standardized deaths rate. (B) Age-standardized DALYs rate. (C) Number of deaths cases. (D) Number of DALYs cases. DALY = disability-adjusted life year.

Similar trends were observed in DALY rates. Burundi and the Democratic Republic of the Congo saw significant increases in DALY rates, while Ghana, Cuba, and Equatorial Guinea showed the largest reductions, with EAPC values of −11.89 (95% CI: −13.31 to −10.45), −8.94 (95% CI: −10.34 to −7.52), and −7.37 (95% CI: −8.5 to −6.22), respectively. Figure 12 and Tables S1–S2 provide further details, Supplemental Digital Content, https://links.lww.com/MD/R530.

### 3.3. Frontier analysis

**Figure 13.**
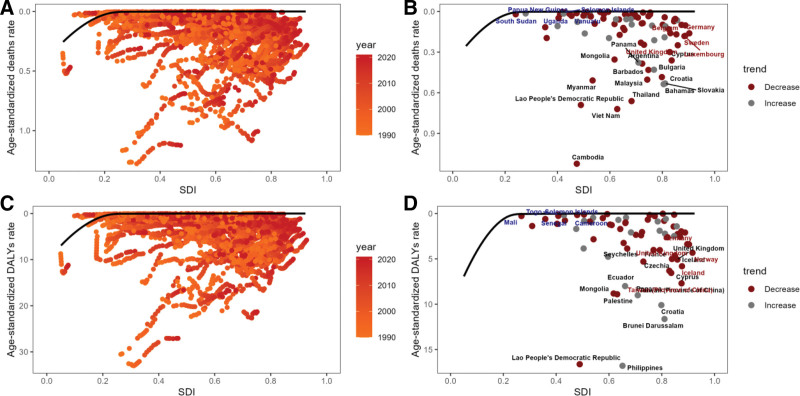
Frontier analysis of colorectal cancer burden attributable to a low-fiber diet by SDI, 1990-2021. (A) Regional trajectories of age-standardized death rates versus SDI. (B) National distribution of age-standardized death rates versus SDI. (C) Regional trajectories of age-standardized DALY rates versus SDI. (D) National distribution of age-standardized DALY rates versus SDI. ASDR = age-standardized death rate, DALYs = disability-adjusted life years, SDI = sociodemographic index.

Figure 13 presents a frontier analysis of ASDR, and DALYs related to CRC associated with low-fiber diets across different SDI regions from 1990 to 2020. This analysis reveals key trends associated with regional differences between countries and the effectiveness of interventions that have been implemented. Figures A and C show the time trajectories of ASDR and DALYs in regions with varying SDI levels. Regions with higher SDI tend to have lower age-standardized death rates and DALYs, which are closely aligned with the frontier and have achieved better outcomes in reducing the burden of childhood cancer. In contrast, regions with lower SDI values exhibit more significant variation, with persistently higher ASDR and DALY rates.

Figure 13B, D illustrate national-level trends, highlighting the performance of specific countries compared to frontier nations. Countries in low-SDI regions, such as Papua New Guinea, South Sudan, Cambodia, and Togo, remain far from the frontier. This indicates higher mortality and DALY rates related to CRC. In comparison, high-SDI countries like Germany, Luxembourg, and Sweden, while still showing some deviation, are closer to the frontier.

### 3.4. Factors influencing EAPC

**Figure 14.**
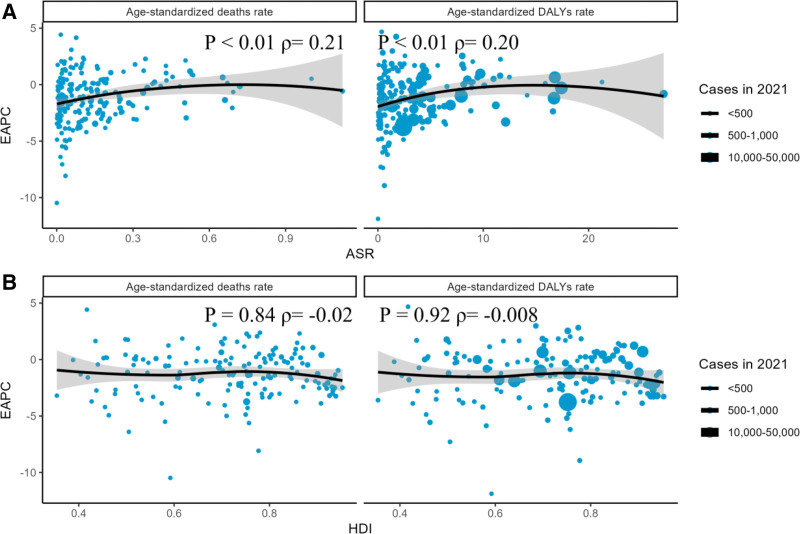
Relationship of EAPCs with ASRs and HDI for colorectal cancer attributable to a low-fiber diet in 2021. (A) Relationship between EAPCs and ASRs for age-standardized death rates and age-standardized DALY rates. (B) Relationship between EAPCs and HDI for age-standardized death rates and age-standardized DALY rates. Circles represent countries with available HDI data; circle size increases with the number of colorectal cancer cases attributable to a low-fiber diet. The rho values and P-values were derived from Spearman correlation analysis. ASR = age-standardized rate, DALY = disability-adjusted life year, EAPC = estimated annual percentage change, HDI = human development index.

In the 2021 study, we explored the correlations between EAPCs and ASDRs as well as the HDI. The analysis revealed a significant positive correlation between EAPCs and ASRs. Specifically, when ASRs remained at lower levels, the positive correlation between EAPCs and ASRs was particularly evident (mortality: Spearman’s correlation coefficient ρ = 0.21, *P*-value < .01; DALYs: ρ = 0.20, *P*-value < .01). However, when ASRs reached higher levels, this positive correlation shifted to a negative correlation (Fig. 14).

## 4. Discussion

In this study, we aimed to quantify the global, regional, and national burden of CRC attributable to low-fiber diets from 1990 to 2021. Our results indicate significant global and regional disparities, with high-income regions showing a decline in CRC burden associated with low-fiber diets, while lower-income regions continue to bear a disproportionate burden. Our findings are consistent with those of previous studies that have examined the impact of dietary patterns on CRC burden. For instance, Xie et al conducted a comprehensive analysis of CRC burden attributable to low-fiber diets from 1990 to 2019 across different regions,^[19]^ which corroborates our findings regarding the global distribution of CRC related to low-fiber intake. Their analysis highlights similar trends in high-income countries, where improvements in dietary habits have led to a reduction in CRC-related mortality, aligning with the results of our study.

Furthermore, the study by Zhu et al provides valuable insights into the temporal trends in CRC burden across 204 countries and territories,^[20]^ offering a more extensive regional analysis of the disease burden attributable to low-fiber diets. Their findings also align with our study’s observation of higher CRC burden in regions with lower socioeconomic development, underscoring the role of socioeconomic factors in shaping dietary patterns and health outcomes.

These studies, along with our own, underscore the importance of public health initiatives aimed at increasing dietary fiber intake, particularly in regions with low socioeconomic development, where the burden of CRC attributable to low-fiber diets remains high. The global decline in CRC mortality and DALYs due to low-fiber diets is particularly pronounced in high-income regions, where public health policies, early detection, and improved healthcare access have played pivotal roles in reducing the burden.^[21-23]^ However, the adoption of Westernized diets in low- and middle-income countries has shifted the burden of CRC to these regions, where CRC incidence and mortality have risen.^[24,25]^

In contrast, high-income countries have seen stabilization or declines in CRC rates, driven by long-term public health initiatives promoting fiber-rich diets and regular screenings.^[26]^ However, low-SDI regions continue to experience disproportionately high CRC burdens, with only slight declines in age-standardized mortality and DALYs. Limited healthcare access, low awareness of CRC risk factors, and financial barriers to healthy eating contribute to this stagnation.^[27]^ The increasing consumption of processed, low-fiber foods exacerbates the problem in these regions.

The frontier analysis revealed persistent disparities between high and low-SDI regions. While high-SDI countries have made significant progress, low-SDI regions face ongoing challenges in managing CRC linked to low-fiber diets.^[28,29]^ These findings underscore the need for targeted public health interventions in low-SDI regions to reduce the global CRC burden.

Despite the established protective role of dietary fiber, global fiber consumption remains below recommended levels, particularly in regions where processed foods are more accessible than fruits, vegetables, and whole grains.^[30,31]^ High-income regions can serve as models for global dietary interventions, including education campaigns, subsidies for healthy foods, and regulations on processed food marketing.^[32,33]^ Tailored community-based programs that promote traditional fiber-rich diets and increase the affordability of fiber-rich foods in low-SDI regions could significantly reduce the CRC burden.^[34,35]^

Governments, non-governmental organizations, and the private sector must collaborate to ensure the sustainability of these interventions and reach the most vulnerable populations.^[36,37]^ Without substantial action, the CRC burden associated with low-fiber diets is expected to persist, particularly in low-SDI regions.^[38]^ Policymakers must prioritize dietary interventions and CRC prevention programs to prevent further exacerbation of disparities in CRC outcomes.^[39]^

This study’s strengths include its global scope, robust statistical methods, and innovative analytical techniques, which allow for the identification of key areas for improvement by comparing regional performance. However, the reliance on self-reported dietary data may introduce bias, and other dietary and lifestyle factors that influence CRC risk were not fully accounted for. Despite these limitations, the findings provide strong support for global and regional initiatives promoting high-fiber diets to reduce the CRC burden.^[40,41]^

## 5. Conclusions

In conclusion, although the attributable burden of CRC associated with a low-fiber diet has exhibited a slight decline on a global scale, notable regional disparities persist. Notable advancements have been observed in high-income regions, largely attributable to the implementation of efficacious public health initiatives, enhanced early detection capabilities, and dietary enhancements. Nevertheless, low-SDI regions continue to experience a disproportionate burden due to restricted access to healthcare, inadequate awareness of CRC risk factors, and the rising prevalence of low-fiber processed diets. To address these inequalities, targeted public health interventions promoting increased fiber intake and improved dietary habits are required, particularly in low SDI areas. These findings should inform the design of future research, and the development of targeted policies aimed at reducing the global and regional burden of CRC and closing the gap between high and low SDI areas.

## Acknowledgments

The authors gratefully acknowledge all participants of the GBD 2021 for their contribution.

## Author contributions

**Conceptualization:** Ming Yin, Anmin Wang, Yuzhou Cai, Yujian Zeng.

**Data curation:** Ming Yin, Anmin Wang, Yuzhou Cai, Yujian Zeng.

**Formal analysis:** Ming Yin, Anmin Wang, Yuzhou Cai, Yujian Zeng.

**Funding acquisition:** Ming Yin, Anmin Wang, Yuzhou Cai, Yujian Zeng.

**Investigation:** Ming Yin, Anmin Wang, Yuzhou Cai, Yujian Zeng.

**Methodology:** Ming Yin, Anmin Wang, Yuzhou Cai, Yujian Zeng.

**Project administration:** Ming Yin, Anmin Wang, Yuzhou Cai, Yujian Zeng.

**Resources:** Anmin Wang, Yuzhou Cai, Yujian Zeng.

**Software:** Ming Yin, Anmin Wang, Yuzhou Cai, Yujian Zeng.

**Supervision:** Ming Yin, Anmin Wang, Yuzhou Cai, Yujian Zeng.

**Validation:** Ming Yin, Anmin Wang, Yuzhou Cai, Yujian Zeng.

**Visualization:** Ming Yin, Anmin Wang, Yuzhou Cai, Yujian Zeng.

**Writing – original draft:** Ming Yin, Anmin Wang, Han Li, Chunyu Yang, Yuzhou Cai, Yujian Zeng.

**Writing – review & editing:** Ming Yin, Anmin Wang, Han Li, Chunyu Yang, Yuzhou Cai.

## Supplementary Material
