# Supplementary material for: Global, regional, and national burden of disease associated with low-fiber dietary patterns for colorectal cancer from 1990 to 2021: A systematic analysis for the global burden of disease 2021
Source: Medicine (Baltimore). 2026 Mar 13;105(11):e47982. doi: 10.1097/MD.0000000000047982 (PMC12991694; doi:10.1097/MD.0000000000047982)
Supplement: Supplementary file 1 [file medi-105-e47982-s001.docx]

**Supplementary Table S1.** Country/territory, GBD region, and SDI–specific deaths and age-standardized mortality rate (ASMR) for colorectal cancer attributable to low-fibre diet in 2021, with 95% uncertainty intervals (UI), and estimated annual percentage change (EAPC) in ASMR from 1990 to 2021, by sex (Both, Male, Female). Rates are per 100,000 population and are age-standardized to the GBD 2021 standard population. ISO3 codes, GBD regions, and SDI quintiles follow GBD taxonomy.

|  | Number of DALYs cases (95% UI) in 1990 | The age-standardized DALYs rate/100000 (95% UI) in 1990 | Number of DALYs cases (95% UI) in 2021 | The age-standardized DALYs rate/100000 (95% UI) in 2021 | EAPC (95% CI) |
| --- | --- | --- | --- | --- | --- |
| Global | 247015 (112606-380299) | 6.17 (2.8-9.47) | 305676 (135089-469863) | 3.58 (1.58-5.5) | -1.89 (-1.95--1.83) |
| Sex |  |  |  |  |  |
| Female | 120347 (54864-184514) | 5.66 (2.58-8.68) | 136096 (60109-210528) | 3 (1.32-4.64) | -2.24 (-2.32--2.17) |
| Male | 126668 (58713-201204) | 6.76 (3.09-10.63) | 169580 (74562-263438) | 4.21 (1.85-6.56) | -1.6 (-1.64--1.55) |
| Age |  |  |  |  |  |
| 25-29 years | 6529 (2770-10688) | 1.47 (0.63-2.41) | 4431 (1929-7094) | 0.75 (0.33-1.21) | -2.49 (-2.6--2.38) |
| 30-34 years | 8496 (3843-12983) | 2.2 (1-3.37) | 8919 (3806-14318) | 1.48 (0.63-2.37) | -1.78 (-2.09--1.47) |
| 35-39 years | 15306 (6769-25352) | 4.35 (1.92-7.2) | 12224 (5260-20410) | 2.18 (0.94-3.64) | -2.45 (-2.59--2.31) |
| 40-44 years | 17941 (7730-28202) | 6.26 (2.7-9.84) | 16707 (7415-26986) | 3.34 (1.48-5.39) | -2.23 (-2.31--2.15) |
| 45-49 years | 20245 (8817-32644) | 8.72 (3.8-14.06) | 23216 (9893-36688) | 4.9 (2.09-7.75) | -1.96 (-2.03--1.9) |
| 50-54 years | 25584 (11770-41837) | 12.04 (5.54-19.68) | 30623 (13665-48003) | 6.88 (3.07-10.79) | -1.91 (-1.96--1.85) |
| 55-59 years | 26843 (12002-42595) | 14.49 (6.48-23) | 33097 (15203-52343) | 8.36 (3.84-13.23) | -1.79 (-1.84--1.75) |
| 60-64 years | 27406 (12842-45126) | 17.06 (8-28.1) | 33094 (14677-52200) | 10.34 (4.59-16.31) | -1.82 (-1.89--1.74) |
| 65-69 years | 26818 (12497-43311) | 21.7 (10.11-35.04) | 34690 (15271-56018) | 12.58 (5.54-20.31) | -1.94 (-2.03--1.86) |
| 70-74 years | 20577 (9245-31373) | 24.31 (10.92-37.06) | 31953 (13866-50007) | 15.52 (6.74-24.29) | -1.69 (-1.83--1.56) |
| 75-79 years | 21453 (9657-33482) | 34.85 (15.69-54.39) | 25775 (11852-40452) | 19.54 (8.99-30.67) | -1.96 (-2.05--1.88) |
| 80-84 years | 17192 (8166-26398) | 48.6 (23.08-74.62) | 24756 (11187-38025) | 28.27 (12.77-43.42) | -1.75 (-1.83--1.67) |
| 85-89 years | 8209 (3780-13058) | 54.33 (25.02-86.41) | 15164 (6745-24056) | 33.17 (14.75-52.61) | -1.5 (-1.59--1.41) |
| 90-94 years | 3642 (1680-5622) | 84.99 (39.19-131.19) | 8147 (3547-13026) | 45.54 (19.83-72.81) | -2.03 (-2.12--1.94) |
| 95+ years | 774 (343-1269) | 76.02 (33.65-124.61) | 2880 (1198-4716) | 52.84 (21.98-86.53) | -1.18 (-1.39--0.97) |
| SDI region |  |  |  |  |  |
| High-middle SDI | 53927 (25121-84667) | 5.39 (2.51-8.34) | 61103 (26584-95508) | 3.21 (1.39-4.98) | -1.93 (-2.1--1.75) |
| High SDI | 79396 (35518-120306) | 7.29 (3.26-11.04) | 81271 (36171-125964) | 4.03 (1.8-6.22) | -1.86 (-1.92--1.8) |
| Low-middle SDI | 31283 (13697-48905) | 4.61 (2.05-7.16) | 49159 (21832-74648) | 3.22 (1.42-4.91) | -1.43 (-1.56--1.29) |
| Low SDI | 4541 (2032-7088) | 1.83 (0.82-2.83) | 8742 (4067-13475) | 1.53 (0.71-2.35) | -0.9 (-1.15--0.65) |
| Middle SDI | 77615 (36217-121305) | 6.75 (3.14-10.53) | 105062 (46819-162532) | 3.83 (1.71-5.93) | -1.98 (-2.05--1.9) |
| GBD region |  |  |  |  |  |
| Advanced Health System | 96091 (42840-146722) | 6 (2.68-9.16) | 106609 (47859-165045) | 3.86 (1.73-5.97) | -1.5 (-1.56--1.44) |
| Africa | 3009 (1360-4707) | 1 (0.45-1.56) | 5375 (2425-8779) | 0.75 (0.34-1.21) | -1.07 (-1.12--1.02) |
| African Region | 2280 (1033-3567) | 0.98 (0.44-1.52) | 3694 (1630-5822) | 0.67 (0.29-1.05) | -1.35 (-1.43--1.27) |
| America | 42149 (19046-63798) | 6.95 (3.14-10.52) | 42380 (19728-65775) | 3.25 (1.51-5.03) | -2.36 (-2.56--2.16) |
| Andean Latin America | 879 (372-1335) | 4.08 (1.73-6.2) | 2068 (885-3192) | 3.42 (1.47-5.29) | -0.37 (-0.55--0.19) |
| Asia | 149015 (70145-234279) | 6.71 (3.16-10.51) | 199582 (87235-307130) | 3.93 (1.72-6.03) | -1.85 (-1.93--1.78) |
| Australasia | 2322 (1042-3666) | 10.04 (4.5-15.83) | 2407 (1108-3959) | 4.73 (2.13-7.72) | -2.93 (-3.19--2.67) |
| Basic Health System | 117476 (55828-186023) | 7.23 (3.43-11.44) | 147408 (64207-228059) | 3.9 (1.7-6.02) | -2.1 (-2.18--2.03) |
| Caribbean | 1409 (625-2172) | 5.34 (2.36-8.24) | 1416 (647-2226) | 2.66 (1.22-4.18) | -2.31 (-2.59--2.03) |
| Central Africa | 197 (87-315) | 0.67 (0.3-1.09) | 953 (389-1675) | 1.29 (0.52-2.27) | 2.33 (1.84-2.83) |
| Central Asia | 2068 (886-3138) | 4.15 (1.76-6.28) | 1569 (716-2395) | 1.83 (0.83-2.8) | -3.63 (-4.13--3.13) |
| Central Europe | 6758 (3064-10414) | 4.63 (2.09-7.13) | 9227 (4267-14068) | 4.3 (1.99-6.54) | -0.56 (-0.93--0.19) |
| Central Latin America | 1585 (693-2373) | 1.77 (0.77-2.65) | 4824 (2188-7621) | 1.9 (0.87-3.01) | 0.47 (0.3-0.64) |
| Central Sub-Saharan Africa | 286 (124-465) | 1.2 (0.52-1.96) | 976 (404-1702) | 1.61 (0.66-2.85) | 1.1 (0.81-1.4) |
| Commonwealth High Income | 13835 (6112-21281) | 9.36 (4.15-14.26) | 10920 (4924-16916) | 4.21 (1.89-6.52) | -2.59 (-2.78--2.39) |
| Commonwealth Low Income | 6321 (2881-10034) | 6.6 (3.01-10.51) | 10375 (4625-17244) | 4.29 (1.92-7.01) | -1.46 (-1.56--1.36) |
| Commonwealth Middle Income | 16744 (7574-26207) | 2.39 (1.07-3.73) | 27111 (12098-40929) | 1.62 (0.72-2.47) | -1.51 (-1.71--1.32) |
| East Asia | 70375 (30971-115057) | 7.3 (3.25-11.78) | 52616 (20941-90261) | 2.54 (1.01-4.35) | -3.54 (-3.62--3.45) |
| East Asia & Pacific - WB | 125904 (59401-197986) | 8.68 (4.09-13.65) | 162351 (70963-251451) | 5.08 (2.22-7.91) | -1.82 (-1.89--1.76) |
| Eastern Africa | 1463 (633-2372) | 1.91 (0.84-3.05) | 2401 (1098-3991) | 1.32 (0.6-2.18) | -1.59 (-1.71--1.47) |
| Eastern Europe | 9280 (4287-14504) | 3.38 (1.56-5.29) | 12242 (5544-18980) | 3.58 (1.65-5.56) | -1 (-1.62--0.37) |
| Eastern Mediterranean Region | 4067 (1843-6320) | 2.09 (0.94-3.23) | 10346 (4786-16413) | 2 (0.91-3.22) | -0.3 (-0.53--0.07) |
| Eastern Sub-Saharan Africa | 1130 (506-1804) | 1.38 (0.63-2.19) | 1910 (844-3109) | 0.99 (0.43-1.6) | -1.46 (-1.6--1.32) |
| Europe | 52415 (23412-79136) | 5.15 (2.3-7.81) | 57782 (26354-87226) | 3.7 (1.68-5.58) | -1.37 (-1.56--1.18) |
| Europe & Central Asia - WB | 53824 (24005-81256) | 5.15 (2.29-7.8) | 58697 (26798-88635) | 3.63 (1.66-5.48) | -1.44 (-1.64--1.25) |
| European Region | 53950 (24063-81455) | 5.13 (2.28-7.77) | 58883 (26884-88939) | 3.61 (1.64-5.45) | -1.45 (-1.65--1.25) |
| High-income Asia Pacific | 8930 (3852-14039) | 4.52 (1.95-7.12) | 22645 (10058-35310) | 5.3 (2.37-8.25) | 0.85 (0.63-1.07) |
| High-income North America | 30202 (13705-45784) | 8.7 (3.94-13.18) | 19658 (9129-30726) | 3.21 (1.5-5.05) | -3.15 (-3.35--2.94) |
| Latin America & Caribbean - WB | 12122 (5379-18373) | 4.45 (1.97-6.74) | 22930 (10383-35134) | 3.24 (1.46-4.96) | -0.92 (-1.03--0.8) |
| Limited Health System | 32238 (14319-50565) | 3.64 (1.64-5.69) | 48338 (21605-73413) | 2.28 (1.02-3.5) | -1.82 (-1.99--1.66) |
| Middle East & North Africa - WB | 1256 (566-1971) | 0.96 (0.44-1.51) | 2919 (1339-4675) | 0.79 (0.36-1.26) | -0.66 (-0.76--0.55) |
| Minimal Health System | 957 (408-1565) | 1.52 (0.66-2.45) | 2981 (1298-5115) | 2.02 (0.89-3.41) | 0.71 (0.28-1.14) |
| North Africa and Middle East | 1892 (846-3064) | 1.03 (0.46-1.66) | 4637 (2101-7565) | 0.92 (0.42-1.51) | -0.54 (-0.65--0.43) |
| North America | 30200 (13704-45780) | 8.7 (3.94-13.18) | 19667 (9132-30742) | 3.21 (1.5-5.05) | -3.14 (-3.35--2.94) |
| Northern Africa | 315 (139-490) | 0.49 (0.21-0.76) | 466 (187-817) | 0.3 (0.12-0.51) | -1.87 (-2.24--1.5) |
| Oceania | 13 (6-22) | 0.4 (0.17-0.65) | 15 (6-26) | 0.18 (0.07-0.32) | -2.53 (-2.84--2.22) |
| Region of the Americas | 42149 (19046-63798) | 6.95 (3.14-10.52) | 42380 (19728-65775) | 3.25 (1.51-5.03) | -2.36 (-2.56--2.16) |
| South-East Asia Region | 45942 (20745-71540) | 5.85 (2.67-9.12) | 72316 (31080-110919) | 3.83 (1.64-5.86) | -1.66 (-1.79--1.53) |
| South Asia | 20327 (9267-32395) | 3.13 (1.44-5.01) | 32597 (14931-49785) | 2.08 (0.95-3.18) | -1.56 (-1.73--1.38) |
| South Asia - WB | 20724 (9452-33002) | 3.1 (1.43-4.95) | 33824 (15374-51708) | 2.1 (0.96-3.23) | -1.5 (-1.69--1.32) |
| Southeast Asia | 44395 (19963-67110) | 15.82 (7.14-23.9) | 84847 (35935-129659) | 12.4 (5.25-18.96) | -0.94 (-1.01--0.87) |
| Southern Africa | 455 (202-706) | 0.97 (0.43-1.49) | 1011 (448-1603) | 0.98 (0.43-1.53) | 0.1 (-0.03-0.23) |
| Southern Latin America | 4323 (1968-6601) | 9.47 (4.32-14.44) | 5922 (2712-9332) | 6.9 (3.18-10.88) | -0.32 (-0.55--0.08) |
| Southern Sub-Saharan Africa | 263 (115-410) | 0.89 (0.39-1.38) | 812 (360-1291) | 1.31 (0.58-2.05) | 1.48 (1.18-1.79) |
| Sub-Saharan Africa - WB | 2686 (1196-4207) | 1.13 (0.51-1.77) | 4878 (2182-7918) | 0.88 (0.39-1.41) | -0.94 (-0.98--0.9) |
| Tropical Latin America | 3978 (1771-6054) | 4.1 (1.82-6.24) | 8761 (3971-13613) | 3.36 (1.52-5.23) | -1.07 (-1.28--0.86) |
| Western Africa | 580 (262-904) | 0.72 (0.32-1.12) | 545 (232-895) | 0.3 (0.13-0.49) | -3 (-3.35--2.65) |
| Western Europe | 35969 (15982-54262) | 6.31 (2.8-9.5) | 35922 (15800-54483) | 3.92 (1.73-5.91) | -1.52 (-1.61--1.43) |
| Western Pacific Region | 96384 (44177-153043) | 7.79 (3.6-12.38) | 114401 (49648-180379) | 4.25 (1.84-6.68) | -2.01 (-2.07--1.95) |
| Western Sub-Saharan Africa | 630 (284-977) | 0.7 (0.32-1.09) | 607 (260-1011) | 0.3 (0.13-0.49) | -2.98 (-3.32--2.65) |
| World Bank High Income | 86365 (38420-131713) | 6.82 (3.03-10.37) | 93294 (41814-144948) | 4.09 (1.84-6.36) | -1.54 (-1.61--1.48) |
| World Bank Low Income | 2680 (1138-4249) | 1.7 (0.74-2.7) | 6432 (2828-10554) | 1.74 (0.77-2.83) | -0.14 (-0.34-0.06) |
| World Bank Lower Middle Income | 58426 (26403-89236) | 5.09 (2.31-7.79) | 102189 (45033-153964) | 3.82 (1.68-5.74) | -1.16 (-1.32--1.01) |
| World Bank Upper Middle Income | 99290 (46099-157387) | 6.14 (2.88-9.75) | 103421 (44007-168159) | 3.01 (1.29-4.91) | -2.57 (-2.68--2.45) |
| Country |  |  |  |  |  |
| Afghanistan | 245 (67-483) | 3.6 (1.04-7.01) | 1038 (344-2004) | 8.01 (2.94-14.88) | 1.77 (0.89-2.67) |
| Albania | 39 (17-63) | 1.91 (0.83-3.02) | 36 (14-62) | 0.87 (0.34-1.53) | -3.65 (-4.12--3.18) |
| Algeria | 153 (70-237) | 1.32 (0.6-2.02) | 123 (49-210) | 0.36 (0.14-0.59) | -4.8 (-5.3--4.3) |
| American Samoa | 0 (0-0) | 0.38 (0.16-0.66) | 0 (0-0) | 0.21 (0.07-0.43) | -2.08 (-2.42--1.73) |
| Andorra | 2 (1-4) | 3.97 (1.39-6.93) | 4 (1-7) | 2.58 (0.96-4.74) | -1.18 (-1.55--0.81) |
| Angola | 127 (54-214) | 2.91 (1.26-4.8) | 91 (38-159) | 0.71 (0.31-1.23) | -5.86 (-6.45--5.27) |
| Antigua and Barbuda | 10 (4-15) | 17.98 (7.93-27.23) | 15 (7-23) | 14.16 (6.55-21.58) | -0.94 (-1.09--0.79) |
| Argentina | 3094 (1429-4750) | 9.75 (4.52-14.93) | 4693 (2132-7441) | 8.49 (3.89-13.47) | 0.43 (0.12-0.75) |
| Armenia | 146 (65-224) | 5.1 (2.28-7.82) | 99 (43-158) | 2.35 (1.02-3.7) | -3.75 (-4.25--3.24) |
| Australia | 2022 (903-3164) | 10.48 (4.68-16.39) | 2027 (926-3352) | 4.76 (2.13-7.75) | -3.21 (-3.53--2.88) |
| Austria | 624 (287-959) | 5.34 (2.46-8.21) | 291 (131-496) | 1.61 (0.73-2.72) | -3.87 (-4.13--3.61) |
| Azerbaijan | 191 (89-301) | 3.51 (1.63-5.53) | 98 (38-164) | 0.9 (0.36-1.5) | -6.19 (-6.87--5.51) |
| Bahamas | 17 (7-25) | 9.93 (4.29-14.93) | 51 (23-82) | 12.48 (5.68-19.75) | 0.66 (0.44-0.89) |
| Bahrain | 1 (1-3) | 0.74 (0.3-1.38) | 4 (1-8) | 0.45 (0.17-0.81) | -1.99 (-2.18--1.8) |
| Bangladesh | 5993 (2704-9597) | 11.6 (5.23-18.54) | 9866 (4416-16430) | 6.89 (3.07-11.4) | -1.81 (-1.92--1.7) |
| Barbados | 28 (12-43) | 9.62 (4.12-14.99) | 44 (18-72) | 8.78 (3.6-14.57) | 0.18 (-0.06-0.41) |
| Belarus | 74 (30-134) | 0.59 (0.24-1.07) | 136 (56-256) | 0.88 (0.36-1.63) | 0.23 (-0.66-1.13) |
| Belgium | 1069 (481-1612) | 7.05 (3.16-10.64) | 773 (347-1257) | 3.29 (1.52-5.32) | -2.24 (-2.37--2.1) |
| Belize | 1 (1-2) | 1.3 (0.58-2) | 4 (2-7) | 1.27 (0.56-2.07) | 0 (-0.32-0.33) |
| Benin | 2 (1-4) | 0.12 (0.05-0.21) | 5 (2-9) | 0.08 (0.03-0.17) | -0.32 (-0.99-0.34) |
| Bermuda | 3 (1-5) | 5.37 (2.35-8.44) | 12 (5-19) | 9 (3.71-14.65) | 2.04 (1.59-2.49) |
| Bhutan | 14 (6-23) | 4.83 (2.06-8.14) | 14 (6-24) | 2.11 (0.87-3.77) | -2.84 (-2.96--2.73) |
| Bolivia (Plurinational State of) | 132 (55-217) | 3.97 (1.68-6.53) | 377 (154-652) | 4.09 (1.71-6.92) | -0.02 (-0.28-0.25) |
| Bosnia and Herzegovina | 18 (7-33) | 0.46 (0.19-0.81) | 25 (9-49) | 0.41 (0.15-0.81) | -0.38 (-0.85-0.1) |
| Botswana | 10 (5-17) | 1.84 (0.8-3.03) | 27 (11-47) | 1.71 (0.71-2.97) | -0.51 (-1.06-0.04) |
| Brazil | 3966 (1765-6037) | 4.19 (1.86-6.38) | 8719 (3950-13563) | 3.42 (1.55-5.33) | -1.09 (-1.3--0.87) |
| Brunei Darussalam | 10 (5-16) | 8.29 (3.59-13.2) | 45 (20-73) | 11.63 (5.06-18.81) | 2.25 (1.9-2.6) |
| Bulgaria | 806 (362-1258) | 6.83 (3.03-10.72) | 1273 (542-2113) | 9.46 (4-15.45) | 0.66 (-0.06-1.38) |
| Burkina Faso | 14 (6-24) | 0.33 (0.13-0.56) | 17 (6-32) | 0.18 (0.07-0.34) | -1.96 (-2.27--1.64) |
| Burundi | 2 (1-4) | 0.08 (0.03-0.16) | 19 (7-36) | 0.36 (0.13-0.65) | 4.68 (3.92-5.44) |
| Cabo Verde | 1 (1-2) | 0.57 (0.27-0.9) | 4 (2-7) | 0.96 (0.39-1.53) | 0.63 (0.02-1.25) |
| Cambodia | 1640 (669-2712) | 32.66 (13.46-53.29) | 3596 (1669-5905) | 27.12 (12.59-44.31) | -0.82 (-0.92--0.73) |
| Cameroon | 16 (7-27) | 0.35 (0.15-0.6) | 22 (8-45) | 0.17 (0.06-0.34) | -3.73 (-4.16--3.29) |
| Canada | 1946 (876-2965) | 6.05 (2.72-9.26) | 1394 (655-2240) | 2.05 (0.98-3.26) | -3.24 (-3.52--2.96) |
| Central African Republic | 18 (7-31) | 1.43 (0.59-2.49) | 36 (14-68) | 1.38 (0.55-2.54) | -0.07 (-0.44-0.3) |
| Chad | 20 (9-33) | 0.69 (0.31-1.16) | 27 (11-48) | 0.44 (0.18-0.77) | -1.42 (-1.67--1.16) |
| Chile | 620 (277-953) | 6.13 (2.74-9.41) | 884 (405-1421) | 3.51 (1.63-5.61) | -1.56 (-1.78--1.34) |
| China | 68081 (29790-111751) | 7.32 (3.23-11.87) | 48100 (18867-84761) | 2.4 (0.94-4.16) | -3.77 (-3.86--3.67) |
| Colombia | 830 (368-1235) | 4.37 (1.93-6.54) | 1776 (788-2831) | 3.25 (1.43-5.18) | -0.3 (-0.8-0.2) |
| Comoros | 3 (1-4) | 1.22 (0.44-1.97) | 9 (3-15) | 1.7 (0.68-2.82) | 1.52 (1.3-1.74) |
| Congo | 43 (18-71) | 3.89 (1.58-6.27) | 136 (58-221) | 4.39 (1.87-7.06) | 0.13 (-0.06-0.32) |
| Cook Islands | 0 (0-0) | 0.12 (0.04-0.26) | 0 (0-0) | 0.03 (0.01-0.06) | -4.86 (-5.11--4.61) |
| Costa Rica | 103 (46-152) | 5.55 (2.5-8.27) | 444 (186-695) | 8.14 (3.43-12.71) | 1.46 (1.26-1.66) |
| Croatia | 582 (258-882) | 10.02 (4.44-15.12) | 884 (410-1416) | 10.1 (4.66-15.89) | -0.09 (-0.29-0.12) |
| Cuba | 698 (318-1080) | 6.73 (3.07-10.45) | 120 (45-220) | 0.61 (0.23-1.13) | -8.94 (-10.34--7.52) |
| Cyprus | 57 (25-90) | 8.02 (3.52-12.61) | 131 (57-207) | 6.55 (2.83-10.34) | -0.16 (-0.38-0.05) |
| Czechia | 1446 (666-2203) | 10.58 (4.83-16.15) | 1318 (561-2136) | 6.32 (2.69-10.03) | -1.91 (-2.23--1.58) |
| C么te d'Ivoire | 3 (1-6) | 0.08 (0.03-0.14) | 4 (1-9) | 0.04 (0.01-0.07) | -3.22 (-3.47--2.96) |
| Democratic People's Republic of Korea | 482 (205-850) | 2.82 (1.22-4.88) | 1418 (606-2566) | 4.27 (1.8-7.67) | 1.64 (1.42-1.87) |
| Democratic Republic of the Congo | 82 (33-143) | 0.51 (0.21-0.89) | 696 (273-1319) | 1.73 (0.67-3.25) | 4.25 (3.37-5.14) |
| Denmark | 447 (203-690) | 5.64 (2.55-8.61) | 407 (182-674) | 3.38 (1.53-5.56) | -2 (-2.22--1.78) |
| Djibouti | 23 (10-38) | 14.63 (6.25-24.03) | 45 (18-83) | 6.43 (2.69-11.39) | -3.51 (-3.84--3.19) |
| Dominica | 1 (0-1) | 1.03 (0.44-1.68) | 1 (0-1) | 0.68 (0.29-1.2) | -1.47 (-2--0.94) |
| Dominican Republic | 167 (74-264) | 4.31 (1.9-6.87) | 312 (142-493) | 3.05 (1.37-4.8) | -1.43 (-1.75--1.1) |
| Ecuador | 227 (100-348) | 4.13 (1.82-6.36) | 1332 (560-2137) | 8 (3.35-12.89) | 2.54 (1.94-3.15) |
| Egypt | 81 (33-140) | 0.26 (0.11-0.44) | 120 (43-227) | 0.18 (0.07-0.33) | -0.68 (-1.24--0.11) |
| El Salvador | 13 (5-21) | 0.4 (0.17-0.65) | 35 (14-62) | 0.56 (0.22-1) | 1.51 (1.33-1.68) |
| Equatorial Guinea | 6 (2-11) | 2.95 (1.16-5.13) | 3 (1-6) | 0.48 (0.16-0.96) | -7.37 (-8.5--6.22) |
| Eritrea | 21 (8-38) | 1.54 (0.6-2.74) | 37 (15-68) | 1.14 (0.47-2.11) | -0.73 (-0.95--0.51) |
| Estonia | 53 (24-85) | 2.64 (1.18-4.2) | 39 (17-70) | 1.44 (0.62-2.53) | -2.98 (-3.35--2.6) |
| Eswatini | 4 (2-7) | 1.42 (0.58-2.42) | 16 (6-29) | 2.56 (1.02-4.51) | 2.55 (1.88-3.23) |
| Ethiopia | 503 (202-846) | 2.47 (1.04-4.09) | 272 (112-482) | 0.61 (0.25-1.07) | -5.57 (-5.93--5.2) |
| Fiji | 7 (3-12) | 1.74 (0.74-2.8) | 5 (2-9) | 0.69 (0.26-1.22) | -2.72 (-3.46--1.99) |
| Finland | 348 (160-543) | 4.93 (2.26-7.71) | 300 (127-501) | 2.27 (0.98-3.76) | -3.02 (-3.26--2.78) |
| France | 6353 (2871-9914) | 7.68 (3.47-11.82) | 6960 (3010-11233) | 5.02 (2.17-8.1) | -1.14 (-1.22--1.05) |
| Gabon | 10 (4-19) | 1.8 (0.66-3.29) | 15 (6-25) | 1.31 (0.55-2.24) | -1.08 (-1.18--0.98) |
| Gambia | 8 (3-12) | 2.03 (0.91-3.14) | 4 (2-8) | 0.43 (0.18-0.72) | -6.01 (-6.88--5.13) |
| Georgia | 91 (40-146) | 1.49 (0.65-2.39) | 139 (59-229) | 2.49 (1.06-4.08) | 1.84 (1.3-2.38) |
| Germany | 6955 (3210-11208) | 5.47 (2.51-8.8) | 6361 (2787-10269) | 3.41 (1.5-5.51) | -2.14 (-2.4--1.89) |
| Ghana | 11 (4-20) | 0.18 (0.07-0.31) | 1 (0-2) | 0.01 (0-0.02) | -11.89 (-13.31--10.45) |
| Greece | 196 (86-316) | 1.33 (0.6-2.18) | 467 (203-793) | 1.9 (0.84-3.11) | 1.24 (0.97-1.51) |
| Greenland | 5 (2-8) | 12.87 (5.34-20.98) | 3 (1-5) | 4.23 (1.69-7.15) | -3.87 (-4.02--3.72) |
| Grenada | 3 (1-5) | 4.04 (1.72-6.32) | 4 (2-7) | 3.87 (1.74-6.32) | -0.11 (-0.26-0.04) |
| Guam | 0 (0-0) | 0.04 (0.01-0.11) | 0 (0-0) | 0.02 (0-0.05) | -2.5 (-2.81--2.19) |
| Guatemala | 9 (4-15) | 0.24 (0.1-0.42) | 55 (22-95) | 0.47 (0.19-0.81) | 1.66 (1.07-2.26) |
| Guinea | 19 (8-31) | 0.57 (0.25-0.93) | 35 (14-62) | 0.57 (0.23-1) | 0.27 (0.01-0.53) |
| Guinea-Bissau | 36 (15-58) | 8.47 (3.52-13.46) | 19 (7-33) | 2.42 (0.95-4.13) | -4.87 (-5.32--4.41) |
| Guyana | 38 (17-60) | 9.32 (4.16-14.61) | 29 (12-46) | 4.38 (1.81-6.99) | -1.87 (-2.33--1.41) |
| Haiti | 88 (35-149) | 2.71 (1.1-4.59) | 298 (126-520) | 3.85 (1.68-6.73) | 0.55 (0.13-0.98) |
| Honduras | 9 (4-16) | 0.4 (0.17-0.68) | 45 (18-80) | 0.67 (0.27-1.17) | 1.65 (1.25-2.05) |
| Hungary | 996 (433-1573) | 6.99 (3.05-11.05) | 1501 (678-2382) | 8.14 (3.6-12.83) | 0.02 (-0.47-0.52) |
| Iceland | 30 (13-46) | 10.53 (4.59-16.5) | 33 (15-54) | 5.82 (2.56-9.31) | -2 (-2.21--1.79) |
| India | 11793 (5439-18256) | 2.19 (1.01-3.43) | 17210 (7657-26287) | 1.36 (0.6-2.06) | -1.9 (-2.18--1.63) |
| Indonesia | 11852 (5203-18634) | 10.62 (4.65-16.77) | 20050 (8609-33214) | 7.91 (3.42-12.87) | -1.02 (-1.17--0.87) |
| Iran (Islamic Republic of) | 359 (161-568) | 1.29 (0.6-2.07) | 515 (215-825) | 0.63 (0.27-1.01) | -1.74 (-2.19--1.29) |
| Iraq | 41 (18-69) | 0.46 (0.2-0.77) | 367 (156-620) | 1.37 (0.58-2.28) | 2.98 (2.17-3.81) |
| Ireland | 147 (65-236) | 3.69 (1.63-5.91) | 154 (65-262) | 1.97 (0.83-3.29) | -1.41 (-1.66--1.16) |
| Israel | 67 (29-113) | 1.42 (0.62-2.39) | 100 (43-174) | 0.81 (0.34-1.4) | -1.95 (-2.12--1.78) |
| Italy | 3589 (1629-5557) | 4.17 (1.9-6.47) | 4678 (2040-7191) | 3.29 (1.4-5.01) | -0.53 (-0.77--0.29) |
| Jamaica | 24 (11-39) | 1.34 (0.59-2.15) | 65 (25-111) | 2.09 (0.81-3.6) | 1.84 (1.53-2.16) |
| Japan | 6446 (2768-10157) | 3.91 (1.68-6.2) | 13424 (6034-20613) | 4.24 (1.89-6.67) | 0.7 (0.52-0.88) |
| Jordan | 55 (25-87) | 3.57 (1.59-5.63) | 164 (66-276) | 1.97 (0.82-3.27) | -2.57 (-2.81--2.33) |
| Kazakhstan | 660 (270-1025) | 4.93 (2.02-7.66) | 376 (174-609) | 2.05 (0.97-3.31) | -3.75 (-4.58--2.92) |
| Kenya | 50 (21-82) | 0.54 (0.23-0.88) | 123 (56-203) | 0.49 (0.22-0.82) | -0.15 (-0.28--0.02) |
| Kiribati | 0 (0-1) | 1.12 (0.46-1.86) | 1 (0-1) | 0.89 (0.37-1.52) | -1.21 (-1.36--1.06) |
| Kuwait | 11 (5-17) | 1.46 (0.65-2.32) | 46 (20-74) | 1.28 (0.54-2.06) | -0.13 (-0.52-0.25) |
| Kyrgyzstan | 107 (47-168) | 3.43 (1.49-5.39) | 64 (28-104) | 1.22 (0.54-1.96) | -4.26 (-4.69--3.83) |
| Lao People's Democratic Republic | 696 (257-1156) | 30.53 (11.4-49.87) | 851 (339-1435) | 16.61 (6.68-28.11) | -2.37 (-2.5--2.23) |
| Latvia | 137 (61-219) | 3.91 (1.73-6.23) | 132 (55-210) | 3.43 (1.44-5.49) | -1.15 (-1.68--0.63) |
| Lebanon | 11 (4-20) | 0.52 (0.2-0.93) | 39 (15-68) | 0.62 (0.25-1.09) | 2.03 (1.46-2.61) |
| Lesotho | 1 (0-2) | 0.14 (0.05-0.26) | 2 (0-3) | 0.14 (0.05-0.3) | 0.54 (0.37-0.72) |
| Liberia | 22 (10-35) | 1.87 (0.87-3) | 83 (34-155) | 3.47 (1.5-6.5) | 1.7 (1.34-2.05) |
| Libya | 26 (12-43) | 1.29 (0.57-2.13) | 131 (56-225) | 2.25 (0.93-3.79) | 1.7 (1.36-2.05) |
| Lithuania | 41 (17-70) | 0.93 (0.39-1.59) | 78 (34-133) | 1.42 (0.6-2.37) | 1.43 (0.88-1.98) |
| Luxembourg | 44 (20-67) | 8.15 (3.73-12.66) | 36 (15-58) | 3.28 (1.38-5.32) | -2.89 (-3.14--2.63) |
| Madagascar | 133 (59-213) | 2.39 (1.05-3.87) | 315 (128-525) | 2.45 (1.01-4.05) | -0.22 (-0.36--0.08) |
| Malawi | 4 (1-7) | 0.09 (0.03-0.17) | 5 (2-10) | 0.06 (0.02-0.12) | -2.82 (-3.41--2.24) |
| Malaysia | 1933 (895-3062) | 19.42 (9.02-30.68) | 3219 (1434-5103) | 11.19 (5-17.72) | -1.83 (-2.16--1.5) |
| Maldives | 4 (2-7) | 4.61 (1.88-7.51) | 10 (4-16) | 2.53 (1.06-4.03) | -2.66 (-3.05--2.27) |
| Mali | 36 (16-57) | 0.89 (0.4-1.41) | 26 (10-45) | 0.28 (0.1-0.49) | -3.89 (-4.47--3.3) |
| Malta | 19 (9-30) | 4.44 (2.02-7.02) | 18 (8-29) | 1.88 (0.8-3.01) | -1.97 (-2.55--1.39) |
| Marshall Islands | 0 (0-0) | 0.76 (0.31-1.38) | 0 (0-0) | 0.57 (0.23-1.16) | -0.89 (-0.98--0.8) |
| Mauritania | 15 (6-23) | 1.47 (0.6-2.3) | 13 (5-24) | 0.62 (0.26-1.11) | -2.64 (-3.08--2.21) |
| Mauritius | 76 (35-113) | 9.78 (4.45-14.51) | 94 (39-147) | 5.38 (2.28-8.42) | -2.77 (-3.35--2.19) |
| Mexico | 101 (43-172) | 0.22 (0.1-0.39) | 594 (255-1050) | 0.46 (0.2-0.8) | 2.83 (2.45-3.2) |
| Micronesia (Federated States of) | 0 (0-1) | 0.88 (0.34-1.66) | 0 (0-1) | 0.64 (0.26-1.24) | -1 (-1.1--0.9) |
| Monaco | 1 (0-2) | 1.48 (0.6-2.71) | 1 (0-2) | 1.11 (0.4-2.01) | -0.67 (-0.91--0.42) |
| Mongolia | 168 (70-269) | 14.73 (6.07-23.48) | 229 (100-360) | 8.82 (3.8-13.94) | -2.32 (-2.55--2.08) |
| Montenegro | 9 (4-15) | 1.49 (0.68-2.4) | 15 (6-25) | 1.59 (0.63-2.62) | -0.87 (-1.46--0.27) |
| Morocco | 14 (5-27) | 0.09 (0.03-0.18) | 32 (11-66) | 0.09 (0.03-0.19) | -0.04 (-0.15-0.08) |
| Mozambique | 46 (20-72) | 0.82 (0.36-1.33) | 37 (15-63) | 0.35 (0.14-0.59) | -2.79 (-3.24--2.33) |
| Myanmar | 6817 (2790-11758) | 26.75 (10.98-45.43) | 6123 (2658-9740) | 12.12 (5.3-19.48) | -3.31 (-3.59--3.02) |
| Namibia | 16 (7-26) | 2.37 (1.06-3.82) | 33 (15-57) | 2.2 (1-3.7) | -0.22 (-0.36--0.08) |
| Nauru | 0 (0-0) | 0.26 (0.08-0.59) | 0 (0-0) | 0.34 (0.11-0.71) | 0.6 (-0.72-1.94) |
| Nepal | 280 (118-488) | 2.63 (1.11-4.52) | 185 (77-316) | 0.77 (0.32-1.32) | -4.21 (-4.85--3.55) |
| Netherlands | 2072 (935-3147) | 10.36 (4.69-15.72) | 1776 (807-2921) | 5.11 (2.33-8.26) | -2.66 (-2.97--2.36) |
| New Zealand | 300 (136-483) | 7.82 (3.54-12.62) | 381 (161-619) | 4.65 (1.98-7.54) | -0.98 (-1.26--0.69) |
| Nicaragua | 24 (11-38) | 1.4 (0.61-2.18) | 54 (23-87) | 1.02 (0.44-1.67) | -1.83 (-2.15--1.52) |
| Niger | 21 (9-34) | 0.71 (0.32-1.19) | 24 (9-46) | 0.3 (0.12-0.54) | -3.6 (-4.06--3.13) |
| Nigeria | 193 (84-330) | 0.43 (0.19-0.73) | 133 (52-232) | 0.14 (0.05-0.24) | -3.84 (-4.28--3.4) |
| Niue | 0 (0-0) | 0.32 (0.12-0.63) | 0 (0-0) | 0.18 (0.06-0.37) | -2.26 (-2.44--2.08) |
| North Macedonia | 45 (20-70) | 2.44 (1.1-3.82) | 71 (29-120) | 2.25 (0.94-3.78) | -0.67 (-1.12--0.22) |
| Northern Mariana Islands | 0 (0-0) | 0.05 (0.01-0.13) | 0 (0-0) | 0.08 (0.02-0.18) | 1.74 (1.21-2.28) |
| Norway | 664 (303-1013) | 10.02 (4.52-15.34) | 457 (205-724) | 4.32 (1.93-6.82) | -3.28 (-3.51--3.05) |
| Oman | 7 (3-12) | 0.9 (0.4-1.5) | 7 (3-12) | 0.29 (0.12-0.48) | -3.37 (-3.92--2.81) |
| Pakistan | 2246 (1018-3535) | 3.76 (1.73-5.91) | 5323 (2345-8735) | 3.86 (1.72-6.21) | 0 (-0.28-0.28) |
| Palau | 0 (0-0) | 0.3 (0.09-0.64) | 0 (0-0) | 0.17 (0.05-0.36) | -1.61 (-1.78--1.43) |
| Palestine | 96 (44-171) | 10.63 (4.87-18.83) | 242 (103-393) | 8.9 (3.91-14.81) | -0.25 (-0.54-0.04) |
| Panama | 133 (59-201) | 8.55 (3.79-12.9) | 398 (171-634) | 9.01 (3.89-14.37) | 0.24 (-0.02-0.5) |
| Papua New Guinea | 3 (1-5) | 0.12 (0.04-0.23) | 6 (2-11) | 0.09 (0.04-0.18) | -1.48 (-1.66--1.3) |
| Paraguay | 12 (5-19) | 0.5 (0.21-0.81) | 42 (17-74) | 0.71 (0.29-1.25) | 1.26 (1.03-1.49) |
| Peru | 519 (222-813) | 4.09 (1.76-6.45) | 359 (157-608) | 1.05 (0.46-1.77) | -5.19 (-5.82--4.55) |
| Philippines | 4908 (2241-7541) | 14.1 (6.36-21.59) | 15192 (6936-23871) | 16.79 (7.71-26.23) | 0.65 (0.45-0.84) |
| Poland | 920 (410-1461) | 2.14 (0.95-3.38) | 1549 (604-2562) | 2.23 (0.89-3.67) | 0.09 (-0.29-0.47) |
| Portugal | 420 (194-673) | 3.26 (1.51-5.17) | 704 (315-1132) | 2.96 (1.33-4.73) | 0.35 (0.01-0.69) |
| Puerto Rico | 164 (72-269) | 4.57 (2.01-7.49) | 201 (85-348) | 3.19 (1.38-5.39) | -1.24 (-1.35--1.13) |
| Qatar | 1 (0-1) | 0.34 (0.12-0.65) | 2 (1-3) | 0.13 (0.04-0.26) | -3.83 (-4.27--3.4) |
| Republic of Korea | 2203 (964-3440) | 7.36 (3.13-11.57) | 8823 (4061-14507) | 9.72 (4.45-16.03) | 0.97 (0.55-1.39) |
| Republic of Moldova | 64 (27-108) | 1.47 (0.61-2.48) | 132 (57-231) | 2.26 (0.99-3.96) | 1.58 (1.13-2.03) |
| Romania | 583 (254-918) | 2.16 (0.93-3.44) | 575 (232-972) | 1.63 (0.67-2.73) | -1.7 (-2.05--1.34) |
| Russian Federation | 7126 (3304-10931) | 4.01 (1.85-6.18) | 9841 (4520-15307) | 4.23 (2-6.5) | -1.06 (-1.7--0.41) |
| Rwanda | 1 (0-2) | 0.03 (0.01-0.08) | 2 (1-5) | 0.03 (0.01-0.08) | -3.59 (-4.77--2.39) |
| Saint Kitts and Nevis | 9 (4-13) | 23.49 (10.51-35.64) | 15 (6-24) | 21.27 (9.27-34.29) | 0.23 (0.07-0.39) |
| Saint Lucia | 3 (1-5) | 3.72 (1.67-5.78) | 13 (6-22) | 5.67 (2.36-9.13) | 1.7 (1.39-2.01) |
| Saint Vincent and the Grenadines | 3 (1-5) | 4.44 (1.95-6.83) | 5 (2-8) | 3.76 (1.69-6.08) | -1.27 (-1.6--0.95) |
| Samoa | 0 (0-0) | 0.08 (0.03-0.16) | 0 (0-0) | 0.07 (0.02-0.14) | -2.71 (-3.47--1.94) |
| San Marino | 2 (1-3) | 4.42 (1.87-7.44) | 2 (1-3) | 2.1 (0.82-3.72) | -1.55 (-2.01--1.08) |
| Sao Tome and Principe | 0 (0-0) | 0.18 (0.06-0.32) | 0 (0-0) | 0.11 (0.04-0.23) | -0.81 (-1.6--0.02) |
| Saudi Arabia | 41 (18-70) | 0.57 (0.25-0.97) | 170 (66-301) | 0.6 (0.23-1.02) | 0.91 (0.54-1.29) |
| Senegal | 63 (27-100) | 1.87 (0.82-2.94) | 19 (7-37) | 0.26 (0.1-0.48) | -7.29 (-8.28--6.28) |
| Serbia | 534 (238-842) | 5.18 (2.34-8.09) | 647 (256-1072) | 4.04 (1.61-6.71) | -1.69 (-2.13--1.25) |
| Seychelles | 6 (3-9) | 10.14 (4.56-15.82) | 6 (3-10) | 5.3 (2.53-8.54) | -1.9 (-2.05--1.75) |
| Sierra Leone | 147 (69-231) | 7.02 (3.25-10.98) | 166 (67-274) | 4.16 (1.71-6.85) | -1.79 (-1.91--1.67) |
| Singapore | 271 (125-434) | 11.59 (5.31-18.54) | 353 (151-575) | 4.18 (1.77-6.84) | -3.3 (-3.49--3.11) |
| Slovakia | 555 (254-861) | 9.37 (4.28-14.56) | 1082 (446-1781) | 11.6 (4.77-19.06) | 0.79 (0.18-1.4) |
| Slovenia | 117 (50-183) | 4.77 (2.04-7.48) | 117 (51-193) | 2.64 (1.17-4.33) | -2.24 (-2.43--2.04) |
| Solomon Islands | 0 (0-1) | 0.18 (0.06-0.36) | 1 (0-2) | 0.19 (0.06-0.38) | -0.06 (-0.26-0.15) |
| Somalia | 240 (99-439) | 8.26 (3.51-14.74) | 787 (334-1409) | 10.97 (4.82-19.2) | 0.52 (0.12-0.92) |
| South Africa | 190 (85-297) | 0.82 (0.37-1.29) | 643 (282-1018) | 1.3 (0.57-2.06) | 1.84 (1.55-2.14) |
| South Sudan | 9 (3-18) | 0.32 (0.11-0.67) | 14 (5-28) | 0.34 (0.12-0.65) | -0.2 (-0.37--0.03) |
| Spain | 1893 (849-2901) | 3.63 (1.63-5.54) | 4262 (1823-6744) | 4.45 (1.93-7.11) | 1.23 (0.92-1.55) |
| Sri Lanka | 148 (68-227) | 1.31 (0.61-1.99) | 179 (68-314) | 0.69 (0.26-1.17) | -2.11 (-2.47--1.75) |
| Sudan | 319 (120-568) | 3.09 (1.18-5.59) | 520 (198-962) | 2.23 (0.85-4.05) | -1.22 (-1.27--1.16) |
| Suriname | 41 (19-63) | 15.82 (7.07-24.04) | 101 (44-161) | 15.88 (6.88-25.06) | 0.11 (-0.17-0.39) |
| Sweden | 1146 (507-1764) | 7.83 (3.5-12.08) | 721 (301-1178) | 3.25 (1.4-5.17) | -3.17 (-3.35--2.98) |
| Switzerland | 709 (309-1085) | 6.88 (3.01-10.44) | 887 (397-1423) | 4.89 (2.15-7.76) | -1.15 (-1.53--0.76) |
| Syrian Arab Republic | 76 (32-122) | 1.26 (0.52-2.02) | 164 (68-289) | 1.28 (0.54-2.22) | -0.94 (-1.4--0.47) |
| Taiwan (Province of China) | 1812 (815-2732) | 10.72 (4.87-16.27) | 3098 (1314-4869) | 7.7 (3.32-11.91) | -0.62 (-0.84--0.4) |
| Tajikistan | 176 (78-270) | 5.89 (2.61-9.02) | 193 (80-317) | 2.84 (1.19-4.67) | -3.39 (-3.78--2.99) |
| Thailand | 8547 (3824-13196) | 22.27 (10-34.82) | 17235 (7118-28835) | 16.71 (6.98-27.7) | -1.2 (-1.4--1) |
| Timor-Leste | 11 (5-18) | 3.15 (1.36-5.11) | 26 (11-42) | 3.02 (1.29-4.87) | -0.44 (-0.78--0.09) |
| Togo | 2 (1-4) | 0.18 (0.07-0.32) | 5 (2-9) | 0.12 (0.04-0.24) | -1.22 (-1.72--0.71) |
| Tokelau | 0 (0-0) | 0.61 (0.24-1.12) | 0 (0-0) | 0.25 (0.08-0.5) | -3.12 (-3.23--3.01) |
| Tonga | 0 (0-0) | 0.37 (0.15-0.67) | 0 (0-0) | 0.24 (0.09-0.45) | -1.36 (-1.45--1.28) |
| Trinidad and Tobago | 58 (26-89) | 6.86 (2.97-10.56) | 75 (32-126) | 4.03 (1.73-6.67) | -2.9 (-3.31--2.49) |
| Tunisia | 27 (11-45) | 0.54 (0.22-0.91) | 45 (18-86) | 0.35 (0.14-0.65) | -2.11 (-2.38--1.84) |
| Turkey | 181 (71-339) | 0.49 (0.19-0.91) | 318 (121-609) | 0.35 (0.13-0.67) | -0.68 (-0.86--0.49) |
| Turkmenistan | 98 (44-149) | 4.59 (2.05-6.99) | 76 (34-129) | 1.71 (0.78-2.93) | -4.07 (-4.53--3.6) |
| Tuvalu | 0 (0-0) | 0.96 (0.39-1.78) | 0 (0-0) | 0.51 (0.18-0.95) | -1.74 (-1.94--1.55) |
| Uganda | 21 (8-37) | 0.31 (0.12-0.56) | 61 (21-109) | 0.36 (0.12-0.64) | 0.14 (-0.07-0.35) |
| Ukraine | 1785 (794-2810) | 2.58 (1.15-4.08) | 1883 (805-3286) | 2.54 (1.08-4.42) | -1.24 (-1.86--0.62) |
| United Arab Emirates | 9 (3-16) | 1.27 (0.48-2.25) | 45 (18-79) | 1.04 (0.39-1.84) | 1.24 (0.63-1.86) |
| United Kingdom | 9084 (3994-14330) | 10.36 (4.57-15.99) | 6366 (2893-10196) | 5.08 (2.28-8.07) | -2.2 (-2.53--1.86) |
| United Republic of Tanzania | 60 (25-103) | 0.53 (0.22-0.91) | 117 (47-207) | 0.42 (0.17-0.74) | -1.12 (-1.33--0.9) |
| United States of America | 28250 (12888-42745) | 8.97 (4.08-13.59) | 18261 (8373-28368) | 3.35 (1.55-5.27) | -3.12 (-3.32--2.92) |
| United States Virgin Islands | 6 (2-9) | 6.83 (2.64-11.21) | 4 (2-7) | 2.62 (1.04-4.49) | -2.83 (-3.22--2.43) |
| Uruguay | 609 (273-914) | 16.03 (7.19-24.2) | 344 (156-551) | 6.29 (2.86-9.89) | -3.31 (-3.54--3.07) |
| Uzbekistan | 431 (188-658) | 3.42 (1.49-5.26) | 294 (124-474) | 0.99 (0.42-1.61) | -5.37 (-6.02--4.72) |
| Vanuatu | 0 (0-1) | 0.49 (0.18-0.94) | 1 (0-1) | 0.28 (0.1-0.53) | -2.64 (-2.95--2.34) |
| Venezuela (Bolivarian Republic of) | 363 (158-542) | 3.39 (1.48-5.02) | 1423 (608-2395) | 4.76 (2.04-7.95) | 0.68 (0.39-0.98) |
| Viet Nam | 7693 (3343-11895) | 18.49 (8.06-28.71) | 18149 (8162-29470) | 17.41 (7.87-28.31) | -0.29 (-0.44--0.14) |
| Yemen | 140 (61-242) | 2.58 (1.12-4.4) | 539 (232-1011) | 3.31 (1.42-6.18) | 0.01 (-0.32-0.34) |
| Zambia | 16 (7-29) | 0.51 (0.21-0.92) | 66 (22-158) | 0.81 (0.28-1.77) | 1.14 (0.61-1.67) |
| Zimbabwe | 41 (18-67) | 1.01 (0.44-1.67) | 90 (35-154) | 1.17 (0.47-2.02) | 0.28 (-0.23-0.79) |

**Supplementary Table S2.** Country/territory, GBD region, and SDI–specific disability-adjusted life years (DALYs) and age-standardized DALY rate (ASDR) for colorectal cancer attributable to low-fibre diet in 2021, with 95% uncertainty intervals (UI), and estimated annual percentage change (EAPC) in ASDR from 1990 to 2021, by sex (Both, Male, Female). Rates are per 100,000 population and are age-standardized to the GBD 2021 standard population. ISO3 codes, GBD regions, and SDI quintiles follow GBD taxonomy.

|  | Number of deaths cases (95% UI) in 1990 | The age-standardized deaths rate/100000 (95% UI) in 1990 | Number of deaths cases (95% UI) in 2021 | The age-standardized deaths rate/100000 (95% UI) in 2021 | EAPC (95% CI) |
| --- | --- | --- | --- | --- | --- |
| Global | 9689 (4410-14808) | 0.27 (0.12-0.42) | 13145 (5762-20265) | 0.16 (0.07-0.24) | -1.86 (-1.9--1.82) |
| Sex |  |  |  |  |  |
| Female | 5007 (2267-7717) | 0.25 (0.11-0.39) | 6268 (2826-9725) | 0.14 (0.06-0.21) | -2.16 (-2.21--2.1) |
| Male | 4683 (2121-7319) | 0.3 (0.13-0.45) | 6876 (3035-10690) | 0.18 (0.08-0.28) | -1.57 (-1.61--1.54) |
| Age |  |  |  |  |  |
| 25-29 years | 102 (44-168) | 0.02 (0.01-0.04) | 69 (30-111) | 0.01 (0.01-0.02) | -2.52 (-2.63--2.4) |
| 30-34 years | 145 (66-221) | 0.04 (0.02-0.06) | 151 (64-241) | 0.02 (0.01-0.04) | -1.81 (-2.13--1.5) |
| 35-39 years | 285 (126-473) | 0.08 (0.04-0.13) | 226 (97-378) | 0.04 (0.02-0.07) | -2.48 (-2.62--2.34) |
| 40-44 years | 368 (158-576) | 0.13 (0.06-0.2) | 339 (151-549) | 0.07 (0.03-0.11) | -2.26 (-2.34--2.18) |
| 45-49 years | 462 (202-745) | 0.2 (0.09-0.32) | 524 (223-826) | 0.11 (0.05-0.17) | -2 (-2.07--1.93) |
| 50-54 years | 657 (302-1075) | 0.31 (0.14-0.51) | 775 (346-1211) | 0.17 (0.08-0.27) | -1.95 (-2.01--1.9) |
| 55-59 years | 783 (350-1241) | 0.42 (0.19-0.67) | 952 (439-1502) | 0.24 (0.11-0.38) | -1.84 (-1.89--1.8) |
| 60-64 years | 925 (433-1521) | 0.58 (0.27-0.95) | 1102 (488-1741) | 0.34 (0.15-0.54) | -1.87 (-1.95--1.79) |
| 65-69 years | 1070 (498-1726) | 0.87 (0.4-1.4) | 1366 (602-2207) | 0.5 (0.22-0.8) | -2 (-2.09--1.9) |
| 70-74 years | 993 (446-1510) | 1.17 (0.53-1.78) | 1522 (661-2377) | 0.74 (0.32-1.15) | -1.73 (-1.87--1.6) |
| 75-79 years | 1292 (583-2023) | 2.1 (0.95-3.29) | 1532 (707-2401) | 1.16 (0.54-1.82) | -2 (-2.09--1.91) |
| 80-84 years | 1319 (624-2034) | 3.73 (1.77-5.75) | 1884 (852-2904) | 2.15 (0.97-3.32) | -1.78 (-1.86--1.7) |
| 85-89 years | 791 (363-1262) | 5.23 (2.4-8.35) | 1449 (645-2293) | 3.17 (1.41-5.01) | -1.54 (-1.63--1.45) |
| 90-94 years | 406 (187-627) | 9.47 (4.37-14.63) | 906 (394-1441) | 5.06 (2.2-8.05) | -2.05 (-2.14--1.96) |
| 95+ years | 92 (41-151) | 9.07 (4.04-14.83) | 350 (144-572) | 6.41 (2.65-10.49) | -1.11 (-1.32--0.91) |
| SDI region |  |  |  |  |  |
| High-middle SDI | 2002 (917-3117) | 0.22 (0.1-0.34) | 2666 (1154-4141) | 0.14 (0.06-0.21) | -1.63 (-1.82--1.45) |
| High SDI | 3927 (1747-5986) | 0.36 (0.16-0.54) | 4450 (1995-6913) | 0.19 (0.09-0.3) | -1.97 (-2.05--1.89) |
| Low-middle SDI | 1043 (467-1622) | 0.18 (0.08-0.28) | 1784 (784-2738) | 0.13 (0.06-0.2) | -1.25 (-1.38--1.11) |
| Low SDI | 153 (68-236) | 0.07 (0.03-0.12) | 298 (138-458) | 0.06 (0.03-0.1) | -0.74 (-0.96--0.53) |
| Middle SDI | 2554 (1182-3972) | 0.26 (0.12-0.41) | 3931 (1746-6090) | 0.15 (0.07-0.24) | -1.9 (-1.97--1.84) |
| GBD region |  |  |  |  |  |
| Advanced Health System | 4695 (2078-7184) | 0.29 (0.13-0.45) | 5759 (2616-8867) | 0.18 (0.08-0.28) | -1.58 (-1.62--1.53) |
| Africa | 107 (49-169) | 0.04 (0.02-0.07) | 190 (85-308) | 0.03 (0.01-0.05) | -1 (-1.04--0.95) |
| African Region | 83 (38-130) | 0.04 (0.02-0.07) | 134 (59-211) | 0.03 (0.01-0.05) | -1.25 (-1.31--1.18) |
| America | 2029 (921-3101) | 0.35 (0.16-0.53) | 1948 (903-3040) | 0.14 (0.07-0.22) | -2.76 (-2.95--2.57) |
| Andean Latin America | 36 (15-55) | 0.19 (0.08-0.29) | 88 (38-137) | 0.15 (0.07-0.24) | -0.47 (-0.64--0.29) |
| Asia | 4946 (2325-7765) | 0.26 (0.12-0.41) | 7856 (3432-12008) | 0.16 (0.07-0.25) | -1.59 (-1.67--1.51) |
| Australasia | 102 (46-162) | 0.44 (0.2-0.7) | 118 (53-195) | 0.21 (0.09-0.34) | -2.9 (-3.15--2.65) |
| Basic Health System | 3883 (1827-6143) | 0.28 (0.13-0.44) | 5534 (2403-8562) | 0.15 (0.07-0.24) | -2.02 (-2.09--1.94) |
| Caribbean | 60 (27-94) | 0.24 (0.11-0.38) | 62 (28-99) | 0.12 (0.05-0.18) | -2.51 (-2.77--2.25) |
| Central Africa | 7 (3-11) | 0.03 (0.01-0.05) | 32 (13-57) | 0.06 (0.02-0.1) | 2.3 (1.82-2.79) |
| Central Asia | 71 (30-107) | 0.15 (0.07-0.23) | 57 (25-88) | 0.07 (0.03-0.12) | -3.19 (-3.65--2.73) |
| Central Europe | 302 (136-466) | 0.21 (0.1-0.33) | 464 (212-711) | 0.2 (0.09-0.31) | -0.54 (-0.91--0.17) |
| Central Latin America | 61 (27-92) | 0.08 (0.03-0.12) | 196 (91-310) | 0.08 (0.04-0.13) | 0.22 (0.08-0.37) |
| Central Sub-Saharan Africa | 10 (4-16) | 0.05 (0.02-0.08) | 33 (14-59) | 0.07 (0.03-0.12) | 1.19 (0.88-1.5) |
| Commonwealth High Income | 679 (299-1068) | 0.44 (0.2-0.69) | 590 (274-920) | 0.2 (0.09-0.31) | -2.5 (-2.69--2.3) |
| Commonwealth Low Income | 217 (100-345) | 0.26 (0.12-0.41) | 391 (176-641) | 0.18 (0.08-0.3) | -1.23 (-1.37--1.09) |
| Commonwealth Middle Income | 551 (248-863) | 0.09 (0.04-0.15) | 969 (430-1481) | 0.07 (0.03-0.1) | -1.37 (-1.55--1.19) |
| East Asia | 2180 (961-3566) | 0.27 (0.12-0.44) | 1942 (799-3318) | 0.1 (0.04-0.16) | -3.43 (-3.53--3.32) |
| East Asia & Pacific - WB | 4185 (1958-6580) | 0.34 (0.16-0.53) | 6528 (2833-10071) | 0.21 (0.09-0.32) | -1.63 (-1.69--1.56) |
| Eastern Africa | 50 (22-80) | 0.08 (0.04-0.13) | 82 (37-135) | 0.06 (0.03-0.09) | -1.45 (-1.57--1.34) |
| Eastern Europe | 375 (173-584) | 0.14 (0.06-0.22) | 567 (261-889) | 0.16 (0.07-0.25) | -0.7 (-1.3--0.09) |
| Eastern Mediterranean Region | 144 (64-223) | 0.09 (0.04-0.14) | 350 (160-565) | 0.08 (0.04-0.13) | -0.31 (-0.52--0.1) |
| Eastern Sub-Saharan Africa | 38 (17-61) | 0.06 (0.03-0.09) | 65 (28-105) | 0.04 (0.02-0.07) | -1.32 (-1.45--1.19) |
| Europe | 2589 (1149-3943) | 0.25 (0.11-0.39) | 3125 (1429-4748) | 0.18 (0.08-0.27) | -1.35 (-1.5--1.2) |
| Europe & Central Asia - WB | 2637 (1169-4014) | 0.25 (0.11-0.38) | 3155 (1444-4795) | 0.18 (0.08-0.27) | -1.39 (-1.54--1.23) |
| European Region | 2643 (1171-4023) | 0.25 (0.11-0.38) | 3165 (1449-4810) | 0.18 (0.08-0.27) | -1.39 (-1.55--1.23) |
| High-income Asia Pacific | 383 (161-599) | 0.21 (0.09-0.33) | 1324 (601-2055) | 0.24 (0.11-0.37) | 0.78 (0.6-0.96) |
| High-income North America | 1534 (704-2359) | 0.43 (0.2-0.65) | 992 (448-1577) | 0.15 (0.07-0.23) | -3.47 (-3.66--3.28) |
| Latin America & Caribbean - WB | 502 (223-758) | 0.21 (0.09-0.32) | 967 (432-1490) | 0.14 (0.06-0.22) | -1.15 (-1.25--1.05) |
| Limited Health System | 1067 (483-1668) | 0.14 (0.07-0.22) | 1740 (777-2674) | 0.09 (0.04-0.14) | -1.63 (-1.78--1.48) |
| Middle East & North Africa - WB | 45 (20-71) | 0.04 (0.02-0.07) | 108 (49-173) | 0.04 (0.02-0.06) | -0.59 (-0.69--0.48) |
| Minimal Health System | 34 (15-55) | 0.07 (0.03-0.1) | 97 (42-163) | 0.08 (0.04-0.14) | 0.63 (0.24-1.01) |
| North Africa and Middle East | 66 (29-106) | 0.04 (0.02-0.07) | 162 (74-267) | 0.04 (0.02-0.06) | -0.51 (-0.61--0.42) |
| North America | 1534 (704-2359) | 0.43 (0.2-0.65) | 992 (449-1578) | 0.15 (0.07-0.23) | -3.47 (-3.66--3.28) |
| Northern Africa | 11 (5-18) | 0.02 (0.01-0.04) | 18 (7-31) | 0.01 (0.01-0.02) | -1.61 (-1.94--1.28) |
| Oceania | 0 (0-1) | 0.02 (0.01-0.03) | 0 (0-1) | 0.01 (0-0.01) | -2.4 (-2.75--2.05) |
| Region of the Americas | 2029 (921-3101) | 0.35 (0.16-0.53) | 1948 (903-3040) | 0.14 (0.07-0.22) | -2.76 (-2.95--2.57) |
| South-East Asia Region | 1525 (698-2383) | 0.23 (0.11-0.36) | 2691 (1141-4135) | 0.16 (0.07-0.25) | -1.51 (-1.63--1.39) |
| South Asia | 664 (307-1067) | 0.12 (0.06-0.2) | 1166 (533-1795) | 0.08 (0.04-0.13) | -1.44 (-1.6--1.28) |
| South Asia - WB | 678 (313-1087) | 0.12 (0.06-0.19) | 1205 (548-1860) | 0.08 (0.04-0.13) | -1.4 (-1.57--1.23) |
| Southeast Asia | 1524 (691-2296) | 0.63 (0.29-0.95) | 3152 (1338-4822) | 0.52 (0.22-0.8) | -0.8 (-0.86--0.73) |
| Southern Africa | 16 (7-25) | 0.04 (0.02-0.07) | 36 (16-57) | 0.04 (0.02-0.07) | 0.05 (-0.06-0.17) |
| Southern Latin America | 198 (89-299) | 0.46 (0.21-0.69) | 284 (127-446) | 0.32 (0.14-0.5) | -0.48 (-0.71--0.25) |
| Southern Sub-Saharan Africa | 10 (4-15) | 0.04 (0.02-0.06) | 30 (13-46) | 0.06 (0.02-0.09) | 1.23 (0.97-1.49) |
| Sub-Saharan Africa - WB | 95 (43-151) | 0.05 (0.02-0.08) | 171 (76-276) | 0.04 (0.02-0.06) | -0.87 (-0.91--0.83) |
| Tropical Latin America | 148 (66-225) | 0.18 (0.08-0.27) | 338 (153-529) | 0.13 (0.06-0.21) | -1.32 (-1.55--1.1) |
| Western Africa | 23 (10-35) | 0.03 (0.01-0.05) | 21 (9-34) | 0.01 (0.01-0.02) | -2.84 (-3.14--2.53) |
| Western Europe | 1900 (838-2902) | 0.32 (0.14-0.48) | 2079 (919-3168) | 0.19 (0.09-0.29) | -1.58 (-1.68--1.49) |
| Western Pacific Region | 3183 (1464-5030) | 0.3 (0.14-0.48) | 4678 (1988-7360) | 0.17 (0.07-0.27) | -1.78 (-1.85--1.71) |
| Western Sub-Saharan Africa | 25 (11-38) | 0.03 (0.01-0.05) | 23 (10-39) | 0.01 (0.01-0.02) | -2.8 (-3.1--2.51) |
| World Bank High Income | 4306 (1901-6594) | 0.33 (0.15-0.51) | 5139 (2336-7936) | 0.19 (0.09-0.3) | -1.67 (-1.75--1.59) |
| World Bank Low Income | 93 (40-147) | 0.07 (0.03-0.11) | 219 (97-355) | 0.07 (0.03-0.12) | -0.16 (-0.34-0.03) |
| World Bank Lower Middle Income | 1967 (895-3009) | 0.2 (0.09-0.3) | 3690 (1629-5545) | 0.16 (0.07-0.23) | -1.03 (-1.19--0.88) |
| World Bank Upper Middle Income | 3313 (1551-5292) | 0.24 (0.11-0.38) | 4081 (1717-6521) | 0.12 (0.05-0.19) | -2.37 (-2.5--2.25) |
| Country |  |  |  |  |  |
| Afghanistan | 9 (3-16) | 0.14 (0.05-0.26) | 31 (12-59) | 0.31 (0.12-0.56) | 1.79 (0.94-2.65) |
| Albania | 2 (1-3) | 0.09 (0.04-0.14) | 2 (1-3) | 0.05 (0.02-0.08) | -3.35 (-3.79--2.91) |
| Algeria | 6 (3-9) | 0.07 (0.03-0.11) | 5 (2-9) | 0.02 (0.01-0.03) | -4.29 (-4.75--3.83) |
| American Samoa | 0 (0-0) | 0.01 (0-0.02) | 0 (0-0) | 0.01 (0-0.02) | -0.96 (-1.22--0.7) |
| Andorra | 0 (0-0) | 0.19 (0.07-0.34) | 0 (0-0) | 0.13 (0.04-0.24) | -1.12 (-1.51--0.73) |
| Angola | 4 (2-7) | 0.12 (0.05-0.2) | 3 (1-5) | 0.03 (0.01-0.06) | -5.51 (-6.08--4.93) |
| Antigua and Barbuda | 0 (0-1) | 0.81 (0.35-1.24) | 1 (0-1) | 0.69 (0.32-1.07) | -0.65 (-0.81--0.48) |
| Argentina | 142 (65-216) | 0.47 (0.21-0.71) | 221 (99-351) | 0.38 (0.17-0.61) | 0.23 (-0.08-0.53) |
| Armenia | 5 (2-8) | 0.2 (0.09-0.31) | 5 (2-7) | 0.11 (0.05-0.17) | -3.04 (-3.53--2.54) |
| Australia | 88 (40-139) | 0.46 (0.21-0.72) | 97 (43-163) | 0.2 (0.09-0.34) | -3.25 (-3.56--2.93) |
| Austria | 33 (16-52) | 0.27 (0.13-0.42) | 17 (7-29) | 0.08 (0.04-0.14) | -3.88 (-4.12--3.64) |
| Azerbaijan | 6 (3-10) | 0.13 (0.06-0.2) | 3 (1-6) | 0.04 (0.01-0.06) | -5.62 (-6.25--4.99) |
| Bahamas | 1 (0-1) | 0.4 (0.17-0.61) | 2 (1-3) | 0.54 (0.25-0.86) | 0.83 (0.58-1.09) |
| Bahrain | 0 (0-0) | 0.04 (0.01-0.07) | 0 (0-0) | 0.02 (0.01-0.04) | -1.72 (-1.97--1.47) |
| Bangladesh | 204 (93-328) | 0.44 (0.21-0.71) | 372 (168-612) | 0.28 (0.13-0.46) | -1.62 (-1.79--1.46) |
| Barbados | 1 (1-2) | 0.44 (0.18-0.69) | 2 (1-4) | 0.43 (0.18-0.72) | 0.37 (0.2-0.54) |
| Belarus | 3 (1-6) | 0.02 (0.01-0.04) | 6 (3-12) | 0.04 (0.02-0.07) | 0.44 (-0.42-1.31) |
| Belgium | 59 (27-90) | 0.37 (0.17-0.56) | 46 (20-78) | 0.17 (0.07-0.28) | -2.24 (-2.37--2.11) |
| Belize | 0 (0-0) | 0.06 (0.03-0.1) | 0 (0-0) | 0.06 (0.03-0.09) | -0.08 (-0.42-0.25) |
| Benin | 0 (0-0) | 0.01 (0-0.01) | 0 (0-0) | 0 (0-0.01) | -0.48 (-1.06-0.1) |
| Bermuda | 0 (0-0) | 0.28 (0.12-0.45) | 1 (0-1) | 0.43 (0.18-0.69) | 1.66 (1.24-2.08) |
| Bhutan | 0 (0-1) | 0.19 (0.08-0.31) | 1 (0-1) | 0.09 (0.04-0.16) | -2.49 (-2.61--2.38) |
| Bolivia (Plurinational State of) | 5 (2-8) | 0.18 (0.08-0.31) | 16 (7-27) | 0.19 (0.08-0.33) | 0.13 (-0.13-0.39) |
| Bosnia and Herzegovina | 1 (0-1) | 0.02 (0.01-0.04) | 1 (0-2) | 0.02 (0.01-0.04) | -0.23 (-0.69-0.24) |
| Botswana | 0 (0-1) | 0.09 (0.04-0.14) | 1 (0-2) | 0.08 (0.03-0.14) | -0.39 (-0.89-0.11) |
| Brazil | 148 (66-224) | 0.18 (0.08-0.28) | 336 (152-526) | 0.14 (0.06-0.21) | -1.35 (-1.58--1.12) |
| Brunei Darussalam | 0 (0-1) | 0.38 (0.16-0.62) | 2 (1-3) | 0.51 (0.22-0.82) | 2.14 (1.77-2.51) |
| Bulgaria | 33 (15-51) | 0.29 (0.13-0.46) | 63 (27-103) | 0.43 (0.18-0.7) | 1.03 (0.29-1.78) |
| Burkina Faso | 1 (0-1) | 0.02 (0.01-0.03) | 1 (0-1) | 0.01 (0-0.02) | -1.88 (-2.16--1.59) |
| Burundi | 0 (0-0) | 0 (0-0.01) | 1 (0-1) | 0.02 (0.01-0.03) | 4.42 (3.73-5.12) |
| Cabo Verde | 0 (0-0) | 0.03 (0.01-0.04) | 0 (0-0) | 0.05 (0.02-0.09) | 0.96 (0.35-1.58) |
| Cambodia | 55 (23-90) | 1.27 (0.53-2.06) | 129 (60-211) | 1.12 (0.52-1.83) | -0.57 (-0.66--0.48) |
| Cameroon | 1 (0-1) | 0.02 (0.01-0.03) | 1 (0-2) | 0.01 (0-0.02) | -3.3 (-3.69--2.92) |
| Canada | 94 (42-146) | 0.29 (0.13-0.45) | 77 (33-134) | 0.1 (0.04-0.17) | -3.2 (-3.47--2.93) |
| Central African Republic | 1 (0-1) | 0.06 (0.03-0.1) | 1 (0-2) | 0.06 (0.02-0.11) | -0.09 (-0.44-0.27) |
| Chad | 1 (0-1) | 0.03 (0.01-0.05) | 1 (0-2) | 0.02 (0.01-0.04) | -1.3 (-1.55--1.06) |
| Chile | 27 (12-42) | 0.3 (0.13-0.46) | 44 (21-74) | 0.17 (0.08-0.28) | -1.52 (-1.7--1.33) |
| China | 2100 (927-3452) | 0.27 (0.12-0.44) | 1739 (693-3024) | 0.09 (0.04-0.15) | -3.74 (-3.85--3.62) |
| Colombia | 32 (14-48) | 0.2 (0.09-0.3) | 75 (33-122) | 0.14 (0.06-0.22) | -0.58 (-1.05--0.1) |
| Comoros | 0 (0-0) | 0.05 (0.02-0.08) | 0 (0-1) | 0.08 (0.03-0.13) | 1.76 (1.57-1.95) |
| Congo | 1 (1-2) | 0.17 (0.07-0.26) | 4 (2-7) | 0.19 (0.08-0.3) | 0.18 (0.02-0.35) |
| Cook Islands | 0 (0-0) | 0.01 (0-0.01) | 0 (0-0) | 0 (0-0) | -4.87 (-5.09--4.66) |
| Costa Rica | 4 (2-6) | 0.25 (0.12-0.38) | 19 (8-30) | 0.34 (0.15-0.54) | 1.24 (1.02-1.46) |
| Croatia | 27 (12-42) | 0.5 (0.22-0.77) | 47 (21-78) | 0.48 (0.22-0.79) | -0.16 (-0.35-0.04) |
| Cuba | 31 (14-48) | 0.3 (0.14-0.48) | 7 (3-13) | 0.03 (0.01-0.06) | -8.08 (-9.28--6.87) |
| Cyprus | 3 (1-4) | 0.47 (0.2-0.76) | 7 (3-11) | 0.36 (0.15-0.57) | -0.33 (-0.54--0.11) |
| Czechia | 69 (31-104) | 0.5 (0.22-0.75) | 68 (30-107) | 0.3 (0.13-0.48) | -1.87 (-2.2--1.53) |
| C么te d'Ivoire | 0 (0-0) | 0 (0-0.01) | 0 (0-0) | 0 (0-0) | -2.96 (-3.16--2.77) |
| Democratic People's Republic of Korea | 16 (7-28) | 0.11 (0.05-0.2) | 52 (22-92) | 0.16 (0.07-0.29) | 1.43 (1.2-1.67) |
| Democratic Republic of the Congo | 3 (1-5) | 0.02 (0.01-0.04) | 24 (9-44) | 0.08 (0.03-0.14) | 4.16 (3.3-5.03) |
| Denmark | 24 (11-37) | 0.27 (0.12-0.43) | 24 (11-41) | 0.18 (0.08-0.3) | -1.68 (-1.92--1.44) |
| Djibouti | 1 (0-1) | 0.61 (0.26-1.01) | 2 (1-3) | 0.3 (0.13-0.51) | -3.05 (-3.34--2.76) |
| Dominica | 0 (0-0) | 0.05 (0.02-0.09) | 0 (0-0) | 0.04 (0.01-0.07) | -1.37 (-1.81--0.93) |
| Dominican Republic | 7 (3-11) | 0.2 (0.09-0.33) | 13 (6-21) | 0.14 (0.06-0.22) | -1.49 (-1.85--1.12) |
| Ecuador | 10 (4-15) | 0.2 (0.09-0.31) | 55 (23-89) | 0.35 (0.15-0.55) | 2.28 (1.7-2.87) |
| Egypt | 2 (1-4) | 0.01 (0-0.02) | 4 (1-8) | 0.01 (0-0.02) | -0.26 (-0.81-0.29) |
| El Salvador | 1 (0-1) | 0.02 (0.01-0.03) | 1 (1-3) | 0.02 (0.01-0.04) | 1.31 (1.14-1.48) |
| Equatorial Guinea | 0 (0-0) | 0.12 (0.05-0.21) | 0 (0-0) | 0.02 (0.01-0.04) | -7.06 (-8.17--5.94) |
| Eritrea | 1 (0-1) | 0.06 (0.02-0.11) | 1 (0-2) | 0.05 (0.02-0.09) | -0.44 (-0.65--0.22) |
| Estonia | 2 (1-4) | 0.12 (0.05-0.18) | 2 (1-4) | 0.07 (0.03-0.13) | -2.43 (-2.76--2.09) |
| Eswatini | 0 (0-0) | 0.06 (0.03-0.11) | 1 (0-1) | 0.11 (0.04-0.19) | 2.23 (1.64-2.83) |
| Ethiopia | 17 (7-29) | 0.11 (0.05-0.18) | 11 (5-20) | 0.03 (0.01-0.06) | -4.93 (-5.26--4.61) |
| Fiji | 0 (0-0) | 0.07 (0.03-0.11) | 0 (0-0) | 0.03 (0.01-0.06) | -2.4 (-3.06--1.73) |
| Finland | 18 (8-28) | 0.24 (0.11-0.38) | 19 (8-32) | 0.12 (0.05-0.21) | -2.57 (-2.86--2.27) |
| France | 351 (163-552) | 0.4 (0.18-0.62) | 422 (183-691) | 0.25 (0.11-0.41) | -1.27 (-1.35--1.19) |
| Gabon | 0 (0-1) | 0.08 (0.03-0.14) | 1 (0-1) | 0.06 (0.02-0.1) | -0.96 (-1.06--0.86) |
| Gambia | 0 (0-0) | 0.09 (0.04-0.13) | 0 (0-0) | 0.02 (0.01-0.04) | -5.47 (-6.25--4.68) |
| Georgia | 3 (2-6) | 0.06 (0.03-0.09) | 6 (3-10) | 0.1 (0.04-0.17) | 2.38 (1.87-2.89) |
| Germany | 385 (181-623) | 0.29 (0.14-0.46) | 356 (151-584) | 0.16 (0.07-0.26) | -2.5 (-2.75--2.25) |
| Ghana | 0 (0-1) | 0.01 (0-0.02) | 0 (0-0) | 0 (0-0) | -10.48 (-11.66--9.29) |
| Greece | 10 (5-17) | 0.07 (0.03-0.12) | 29 (13-49) | 0.1 (0.04-0.17) | 1.03 (0.76-1.3) |
| Greenland | 0 (0-0) | 0.57 (0.24-0.96) | 0 (0-0) | 0.19 (0.08-0.32) | -3.83 (-3.99--3.67) |
| Grenada | 0 (0-0) | 0.18 (0.08-0.28) | 0 (0-0) | 0.19 (0.08-0.31) | 0.23 (-0.02-0.48) |
| Guam | 0 (0-0) | 0 (0-0.01) | 0 (0-0) | 0 (0-0) | -3.45 (-3.83--3.08) |
| Guatemala | 0 (0-1) | 0.01 (0.01-0.02) | 2 (1-4) | 0.02 (0.01-0.03) | 0.99 (0.36-1.63) |
| Guinea | 1 (0-1) | 0.03 (0.01-0.04) | 1 (1-2) | 0.03 (0.01-0.04) | 0.16 (-0.06-0.38) |
| Guinea-Bissau | 1 (1-2) | 0.35 (0.14-0.54) | 1 (0-1) | 0.12 (0.04-0.2) | -4.2 (-4.62--3.79) |
| Guyana | 1 (1-2) | 0.4 (0.18-0.63) | 1 (0-2) | 0.19 (0.08-0.31) | -1.91 (-2.32--1.49) |
| Haiti | 3 (1-5) | 0.13 (0.05-0.22) | 11 (5-19) | 0.17 (0.07-0.31) | 0.43 (0.04-0.82) |
| Honduras | 0 (0-1) | 0.02 (0.01-0.03) | 2 (1-3) | 0.03 (0.01-0.05) | 1.86 (1.47-2.25) |
| Hungary | 47 (20-74) | 0.33 (0.14-0.52) | 72 (31-114) | 0.35 (0.16-0.56) | -0.27 (-0.76-0.23) |
| Iceland | 1 (1-2) | 0.49 (0.21-0.76) | 2 (1-3) | 0.29 (0.13-0.47) | -1.69 (-1.93--1.44) |
| India | 368 (169-579) | 0.08 (0.04-0.13) | 605 (264-925) | 0.05 (0.02-0.08) | -1.67 (-1.93--1.42) |
| Indonesia | 387 (169-612) | 0.42 (0.18-0.66) | 712 (310-1166) | 0.34 (0.15-0.54) | -0.71 (-0.86--0.56) |
| Iran (Islamic Republic of) | 12 (6-19) | 0.06 (0.03-0.09) | 21 (9-33) | 0.03 (0.01-0.05) | -1.49 (-1.92--1.05) |
| Iraq | 1 (1-2) | 0.02 (0.01-0.03) | 13 (5-21) | 0.06 (0.02-0.1) | 3.09 (2.33-3.85) |
| Ireland | 7 (3-12) | 0.19 (0.08-0.3) | 8 (3-14) | 0.1 (0.04-0.17) | -1.34 (-1.6--1.07) |
| Israel | 3 (1-6) | 0.08 (0.03-0.13) | 6 (2-10) | 0.04 (0.02-0.08) | -2.08 (-2.25--1.92) |
| Italy | 179 (80-279) | 0.2 (0.09-0.31) | 271 (116-418) | 0.16 (0.07-0.24) | -0.47 (-0.74--0.21) |
| Jamaica | 1 (1-2) | 0.07 (0.03-0.12) | 3 (1-5) | 0.1 (0.04-0.17) | 1.57 (1.3-1.85) |
| Japan | 291 (122-453) | 0.18 (0.08-0.29) | 825 (360-1337) | 0.18 (0.08-0.29) | 0.41 (0.26-0.56) |
| Jordan | 2 (1-3) | 0.15 (0.07-0.23) | 6 (2-10) | 0.09 (0.04-0.15) | -2.3 (-2.5--2.1) |
| Kazakhstan | 23 (9-36) | 0.19 (0.08-0.29) | 14 (7-23) | 0.09 (0.04-0.14) | -3.34 (-4.13--2.53) |
| Kenya | 2 (1-3) | 0.02 (0.01-0.04) | 4 (2-7) | 0.02 (0.01-0.04) | 0.22 (0.05-0.39) |
| Kiribati | 0 (0-0) | 0.05 (0.02-0.08) | 0 (0-0) | 0.04 (0.02-0.06) | -1.11 (-1.25--0.97) |
| Kuwait | 0 (0-1) | 0.07 (0.03-0.11) | 2 (1-3) | 0.06 (0.02-0.1) | 0.05 (-0.39-0.49) |
| Kyrgyzstan | 4 (2-6) | 0.13 (0.06-0.2) | 2 (1-3) | 0.05 (0.02-0.08) | -3.89 (-4.34--3.45) |
| Lao People's Democratic Republic | 23 (9-38) | 1.17 (0.45-1.89) | 30 (12-50) | 0.69 (0.29-1.17) | -2.06 (-2.18--1.94) |
| Latvia | 6 (3-10) | 0.17 (0.07-0.27) | 7 (3-11) | 0.16 (0.07-0.26) | -0.74 (-1.29--0.19) |
| Lebanon | 0 (0-1) | 0.03 (0.01-0.05) | 2 (1-4) | 0.03 (0.01-0.05) | 2.05 (1.47-2.62) |
| Lesotho | 0 (0-0) | 0.01 (0-0.01) | 0 (0-0) | 0.01 (0-0.01) | 0.28 (0.12-0.44) |
| Liberia | 1 (0-1) | 0.09 (0.04-0.14) | 3 (1-6) | 0.16 (0.07-0.29) | 1.64 (1.3-1.98) |
| Libya | 1 (0-2) | 0.06 (0.02-0.1) | 5 (2-8) | 0.1 (0.04-0.17) | 1.87 (1.55-2.19) |
| Lithuania | 2 (1-3) | 0.04 (0.02-0.07) | 4 (2-7) | 0.06 (0.03-0.11) | 1.48 (0.95-2.02) |
| Luxembourg | 2 (1-3) | 0.41 (0.19-0.63) | 2 (1-4) | 0.18 (0.07-0.31) | -2.54 (-2.79--2.28) |
| Madagascar | 5 (2-7) | 0.1 (0.04-0.16) | 10 (4-17) | 0.11 (0.04-0.18) | 0.01 (-0.13-0.15) |
| Malawi | 0 (0-0) | 0 (0-0.01) | 0 (0-0) | 0 (0-0.01) | -2.68 (-3.22--2.13) |
| Malaysia | 72 (33-113) | 0.81 (0.38-1.28) | 129 (58-202) | 0.5 (0.22-0.8) | -1.61 (-1.82--1.41) |
| Maldives | 0 (0-0) | 0.2 (0.08-0.32) | 0 (0-1) | 0.12 (0.05-0.2) | -2.2 (-2.58--1.82) |
| Mali | 1 (1-2) | 0.04 (0.02-0.07) | 1 (0-2) | 0.01 (0.01-0.02) | -3.71 (-4.26--3.16) |
| Malta | 1 (0-1) | 0.22 (0.1-0.36) | 1 (0-2) | 0.1 (0.04-0.16) | -1.84 (-2.39--1.3) |
| Marshall Islands | 0 (0-0) | 0.03 (0.01-0.06) | 0 (0-0) | 0.02 (0.01-0.05) | -0.91 (-0.99--0.83) |
| Mauritania | 1 (0-1) | 0.07 (0.03-0.1) | 1 (0-1) | 0.03 (0.01-0.06) | -2.38 (-2.8--1.95) |
| Mauritius | 3 (1-4) | 0.41 (0.18-0.61) | 4 (2-6) | 0.23 (0.1-0.36) | -2.62 (-3.14--2.09) |
| Mexico | 4 (2-7) | 0.01 (0-0.02) | 23 (10-39) | 0.02 (0.01-0.03) | 2.06 (1.66-2.47) |
| Micronesia (Federated States of) | 0 (0-0) | 0.04 (0.01-0.07) | 0 (0-0) | 0.03 (0.01-0.05) | -0.97 (-1.06--0.88) |
| Monaco | 0 (0-0) | 0.07 (0.03-0.13) | 0 (0-0) | 0.05 (0.02-0.1) | -0.61 (-0.85--0.38) |
| Mongolia | 6 (2-9) | 0.55 (0.23-0.88) | 8 (3-12) | 0.35 (0.15-0.56) | -2.07 (-2.28--1.86) |
| Montenegro | 0 (0-1) | 0.07 (0.03-0.11) | 1 (0-1) | 0.08 (0.03-0.14) | -0.33 (-0.86-0.21) |
| Morocco | 1 (0-1) | 0 (0-0.01) | 1 (0-3) | 0 (0-0.01) | -0.16 (-0.28--0.04) |
| Mozambique | 2 (1-3) | 0.04 (0.02-0.07) | 1 (1-2) | 0.02 (0.01-0.03) | -2.74 (-3.17--2.31) |
| Myanmar | 228 (94-385) | 1.02 (0.43-1.73) | 229 (102-368) | 0.51 (0.23-0.83) | -2.95 (-3.21--2.68) |
| Namibia | 1 (0-1) | 0.1 (0.04-0.16) | 1 (1-2) | 0.09 (0.04-0.15) | -0.13 (-0.25--0.02) |
| Nauru | 0 (0-0) | 0.01 (0-0.02) | 0 (0-0) | 0.01 (0-0.03) | 0.6 (-0.7-1.91) |
| Nepal | 9 (4-15) | 0.1 (0.04-0.18) | 7 (3-12) | 0.03 (0.01-0.06) | -3.89 (-4.51--3.25) |
| Netherlands | 104 (46-159) | 0.5 (0.22-0.77) | 99 (43-164) | 0.26 (0.11-0.42) | -2.42 (-2.69--2.14) |
| New Zealand | 14 (6-22) | 0.36 (0.15-0.58) | 21 (9-34) | 0.23 (0.1-0.38) | -0.66 (-0.94--0.38) |
| Nicaragua | 1 (0-1) | 0.06 (0.03-0.1) | 2 (1-3) | 0.04 (0.02-0.07) | -1.79 (-2.08--1.49) |
| Niger | 1 (0-1) | 0.03 (0.01-0.05) | 1 (0-2) | 0.01 (0.01-0.03) | -3.2 (-3.63--2.76) |
| Nigeria | 8 (3-13) | 0.02 (0.01-0.03) | 5 (2-9) | 0.01 (0-0.01) | -3.57 (-3.97--3.16) |
| Niue | 0 (0-0) | 0.01 (0.01-0.03) | 0 (0-0) | 0.01 (0-0.02) | -2.14 (-2.31--1.97) |
| North Macedonia | 2 (1-3) | 0.11 (0.05-0.18) | 3 (1-6) | 0.12 (0.05-0.2) | -0.37 (-0.9-0.16) |
| Northern Mariana Islands | 0 (0-0) | 0 (0-0.01) | 0 (0-0) | 0 (0-0.01) | 1.75 (1.21-2.29) |
| Norway | 35 (16-54) | 0.48 (0.22-0.74) | 29 (13-48) | 0.25 (0.11-0.41) | -2.48 (-2.65--2.31) |
| Oman | 0 (0-0) | 0.04 (0.02-0.06) | 0 (0-0) | 0.01 (0.01-0.02) | -3.02 (-3.66--2.38) |
| Pakistan | 82 (38-130) | 0.16 (0.07-0.25) | 182 (81-293) | 0.16 (0.07-0.26) | 0.09 (-0.17-0.35) |
| Palau | 0 (0-0) | 0.01 (0-0.03) | 0 (0-0) | 0.01 (0-0.02) | -1.36 (-1.54--1.17) |
| Palestine | 4 (2-6) | 0.47 (0.21-0.8) | 9 (4-15) | 0.41 (0.18-0.66) | -0.13 (-0.46-0.2) |
| Panama | 5 (2-8) | 0.37 (0.16-0.56) | 17 (7-26) | 0.38 (0.16-0.59) | 0.09 (-0.19-0.36) |
| Papua New Guinea | 0 (0-0) | 0 (0-0.01) | 0 (0-0) | 0 (0-0.01) | -1.16 (-1.34--0.98) |
| Paraguay | 0 (0-1) | 0.02 (0.01-0.04) | 2 (1-3) | 0.03 (0.01-0.06) | 1.36 (1.14-1.58) |
| Peru | 21 (9-33) | 0.18 (0.08-0.29) | 17 (7-30) | 0.05 (0.02-0.09) | -4.87 (-5.44--4.31) |
| Philippines | 157 (71-241) | 0.57 (0.26-0.88) | 518 (239-810) | 0.65 (0.3-1.02) | 0.65 (0.47-0.82) |
| Poland | 42 (18-66) | 0.1 (0.04-0.16) | 82 (32-139) | 0.11 (0.04-0.19) | 0.17 (-0.23-0.56) |
| Portugal | 22 (10-34) | 0.17 (0.08-0.27) | 42 (18-71) | 0.14 (0.07-0.24) | 0.11 (-0.17-0.4) |
| Puerto Rico | 7 (3-12) | 0.21 (0.09-0.34) | 10 (4-17) | 0.13 (0.06-0.23) | -1.52 (-1.62--1.41) |
| Qatar | 0 (0-0) | 0.02 (0.01-0.03) | 0 (0-0) | 0.01 (0-0.01) | -3.75 (-4.26--3.24) |
| Republic of Korea | 82 (35-128) | 0.37 (0.17-0.57) | 481 (218-807) | 0.52 (0.24-0.88) | 1.19 (0.82-1.56) |
| Republic of Moldova | 3 (1-4) | 0.06 (0.03-0.1) | 6 (2-10) | 0.09 (0.04-0.16) | 1.47 (1.08-1.86) |
| Romania | 23 (10-37) | 0.09 (0.04-0.14) | 28 (11-48) | 0.07 (0.03-0.12) | -1.39 (-1.73--1.05) |
| Russian Federation | 287 (132-441) | 0.17 (0.08-0.26) | 459 (215-721) | 0.19 (0.09-0.3) | -0.74 (-1.36--0.11) |
| Rwanda | 0 (0-0) | 0 (0-0) | 0 (0-0) | 0 (0-0) | -2.99 (-4.04--1.93) |
| Saint Kitts and Nevis | 0 (0-1) | 1.04 (0.47-1.6) | 1 (0-1) | 1 (0.45-1.59) | 0.52 (0.33-0.71) |
| Saint Lucia | 0 (0-0) | 0.19 (0.08-0.31) | 1 (0-1) | 0.26 (0.11-0.43) | 1.15 (0.9-1.4) |
| Saint Vincent and the Grenadines | 0 (0-0) | 0.22 (0.1-0.34) | 0 (0-0) | 0.17 (0.08-0.28) | -1.39 (-1.67--1.11) |
| Samoa | 0 (0-0) | 0 (0-0.01) | 0 (0-0) | 0 (0-0.01) | -2.73 (-3.42--2.05) |
| San Marino | 0 (0-0) | 0.23 (0.09-0.38) | 0 (0-0) | 0.1 (0.04-0.18) | -1.65 (-2.16--1.14) |
| Sao Tome and Principe | 0 (0-0) | 0.01 (0-0.02) | 0 (0-0) | 0.01 (0-0.01) | -0.55 (-1.22-0.13) |
| Saudi Arabia | 1 (1-2) | 0.03 (0.01-0.04) | 5 (2-8) | 0.02 (0.01-0.04) | 0.52 (0.17-0.86) |
| Senegal | 2 (1-4) | 0.08 (0.04-0.13) | 1 (0-2) | 0.01 (0.01-0.03) | -6.4 (-7.3--5.5) |
| Serbia | 23 (10-36) | 0.25 (0.11-0.39) | 33 (13-56) | 0.19 (0.08-0.33) | -1.67 (-2.06--1.28) |
| Seychelles | 0 (0-0) | 0.42 (0.19-0.68) | 0 (0-0) | 0.25 (0.12-0.41) | -1.52 (-1.66--1.39) |
| Sierra Leone | 6 (3-9) | 0.31 (0.14-0.49) | 6 (3-11) | 0.2 (0.08-0.33) | -1.59 (-1.69--1.48) |
| Singapore | 10 (5-17) | 0.52 (0.23-0.85) | 17 (7-28) | 0.21 (0.09-0.34) | -3.01 (-3.18--2.83) |
| Slovakia | 24 (11-38) | 0.41 (0.19-0.64) | 51 (21-82) | 0.53 (0.22-0.86) | 0.91 (0.33-1.51) |
| Slovenia | 6 (2-9) | 0.22 (0.1-0.36) | 7 (3-11) | 0.14 (0.06-0.23) | -1.91 (-2.09--1.73) |
| Solomon Islands | 0 (0-0) | 0.01 (0-0.02) | 0 (0-0) | 0.01 (0-0.02) | -0.29 (-0.47--0.1) |
| Somalia | 7 (3-13) | 0.33 (0.14-0.59) | 25 (11-44) | 0.46 (0.2-0.8) | 0.69 (0.31-1.07) |
| South Africa | 7 (3-11) | 0.04 (0.02-0.06) | 24 (11-38) | 0.06 (0.02-0.09) | 1.55 (1.29-1.81) |
| South Sudan | 0 (0-1) | 0.01 (0.01-0.03) | 0 (0-1) | 0.01 (0.01-0.03) | -0.04 (-0.16-0.09) |
| Spain | 98 (44-154) | 0.18 (0.08-0.29) | 241 (103-400) | 0.21 (0.09-0.34) | 1.06 (0.78-1.35) |
| Sri Lanka | 6 (3-8) | 0.06 (0.03-0.09) | 8 (3-13) | 0.03 (0.01-0.05) | -2.13 (-2.44--1.83) |
| Sudan | 11 (4-20) | 0.12 (0.05-0.22) | 17 (7-31) | 0.09 (0.04-0.16) | -1.1 (-1.16--1.04) |
| Suriname | 2 (1-2) | 0.68 (0.3-1.03) | 4 (2-7) | 0.67 (0.29-1.1) | 0.15 (-0.14-0.43) |
| Sweden | 62 (29-97) | 0.38 (0.18-0.6) | 46 (19-77) | 0.18 (0.07-0.3) | -2.76 (-2.95--2.57) |
| Switzerland | 36 (16-57) | 0.33 (0.14-0.51) | 51 (22-84) | 0.24 (0.11-0.4) | -0.96 (-1.29--0.62) |
| Syrian Arab Republic | 3 (1-4) | 0.05 (0.02-0.08) | 6 (3-11) | 0.06 (0.02-0.1) | -0.43 (-0.82--0.04) |
| Taiwan (Province of China) | 64 (29-98) | 0.44 (0.2-0.67) | 152 (65-239) | 0.36 (0.15-0.56) | -0.23 (-0.44--0.02) |
| Tajikistan | 6 (3-9) | 0.22 (0.1-0.34) | 6 (3-10) | 0.11 (0.05-0.18) | -3.08 (-3.49--2.66) |
| Thailand | 306 (139-481) | 0.93 (0.43-1.46) | 704 (296-1186) | 0.66 (0.28-1.11) | -1.36 (-1.56--1.17) |
| Timor-Leste | 0 (0-1) | 0.14 (0.06-0.22) | 1 (0-2) | 0.13 (0.06-0.22) | -0.32 (-0.63--0.02) |
| Togo | 0 (0-0) | 0.01 (0-0.02) | 0 (0-0) | 0.01 (0-0.01) | -1.15 (-1.61--0.69) |
| Tokelau | 0 (0-0) | 0.03 (0.01-0.05) | 0 (0-0) | 0.01 (0-0.02) | -3.06 (-3.17--2.95) |
| Tonga | 0 (0-0) | 0.02 (0.01-0.03) | 0 (0-0) | 0.01 (0-0.02) | -1.18 (-1.27--1.09) |
| Trinidad and Tobago | 2 (1-4) | 0.33 (0.14-0.51) | 3 (1-5) | 0.18 (0.08-0.28) | -3.03 (-3.39--2.68) |
| Tunisia | 1 (0-2) | 0.03 (0.01-0.04) | 2 (1-4) | 0.02 (0.01-0.03) | -2.11 (-2.35--1.88) |
| Turkey | 7 (3-12) | 0.02 (0.01-0.04) | 14 (5-27) | 0.02 (0.01-0.03) | -0.25 (-0.44--0.06) |
| Turkmenistan | 3 (1-5) | 0.17 (0.08-0.26) | 2 (1-4) | 0.06 (0.03-0.11) | -4.05 (-4.49--3.6) |
| Tuvalu | 0 (0-0) | 0.04 (0.02-0.07) | 0 (0-0) | 0.02 (0.01-0.04) | -1.66 (-1.84--1.47) |
| Uganda | 1 (0-1) | 0.01 (0.01-0.03) | 2 (1-4) | 0.02 (0.01-0.03) | 0.06 (-0.12-0.24) |
| Ukraine | 72 (32-114) | 0.1 (0.05-0.16) | 82 (35-143) | 0.1 (0.04-0.18) | -1.05 (-1.64--0.45) |
| United Arab Emirates | 0 (0-0) | 0.06 (0.02-0.1) | 1 (0-2) | 0.05 (0.02-0.1) | 2.31 (1.58-3.05) |
| United Kingdom | 463 (204-750) | 0.49 (0.22-0.79) | 359 (164-577) | 0.25 (0.11-0.4) | -2.07 (-2.4--1.75) |
| United Republic of Tanzania | 2 (1-4) | 0.02 (0.01-0.04) | 4 (2-8) | 0.02 (0.01-0.03) | -0.87 (-1.1--0.63) |
| United States of America | 1440 (663-2216) | 0.44 (0.2-0.67) | 914 (407-1443) | 0.15 (0.07-0.24) | -3.46 (-3.65--3.27) |
| United States Virgin Islands | 0 (0-0) | 0.32 (0.12-0.53) | 0 (0-0) | 0.11 (0.05-0.19) | -3.03 (-3.41--2.66) |
| Uruguay | 29 (13-44) | 0.75 (0.34-1.13) | 20 (8-32) | 0.31 (0.14-0.5) | -3.09 (-3.29--2.9) |
| Uzbekistan | 14 (6-22) | 0.12 (0.05-0.19) | 10 (4-16) | 0.04 (0.02-0.06) | -4.99 (-5.57--4.41) |
| Vanuatu | 0 (0-0) | 0.02 (0.01-0.04) | 0 (0-0) | 0.01 (0-0.02) | -2.39 (-2.64--2.14) |
| Venezuela (Bolivarian Republic of) | 14 (6-20) | 0.15 (0.06-0.22) | 56 (25-94) | 0.2 (0.09-0.33) | 0.49 (0.19-0.79) |
| Viet Nam | 284 (125-439) | 0.72 (0.32-1.11) | 683 (306-1113) | 0.72 (0.32-1.17) | -0.14 (-0.31-0.04) |
| Yemen | 5 (2-8) | 0.11 (0.05-0.18) | 18 (8-34) | 0.14 (0.06-0.26) | 0.13 (-0.18-0.45) |
| Zambia | 1 (0-1) | 0.02 (0.01-0.04) | 2 (1-5) | 0.04 (0.01-0.07) | 1.24 (0.73-1.76) |
| Zimbabwe | 2 (1-3) | 0.05 (0.02-0.08) | 3 (1-5) | 0.05 (0.02-0.09) | 0.01 (-0.41-0.43) |
